# Maize RNA 3'-terminal phosphate cyclase-like protein promotes 18S pre-rRNA cleavage and is important for kernel development

**DOI:** 10.1093/plcell/koac052

**Published:** 2022-02-15

**Authors:** Tao Wang, Yumei Chang, Kai Zhao, Qing Dong, Jun Yang

**Affiliations:** School of Life Sciences, The National Engineering Laboratory of Crop Resistance Breeding, Anhui Agricultural University, Hefei 230036, China; School of Life Sciences, The National Engineering Laboratory of Crop Resistance Breeding, Anhui Agricultural University, Hefei 230036, China; School of Life Sciences, The National Engineering Laboratory of Crop Resistance Breeding, Anhui Agricultural University, Hefei 230036, China; Anhui Academy of Agricultural Sciences, Hefei 230031, China; School of Life Sciences, The National Engineering Laboratory of Crop Resistance Breeding, Anhui Agricultural University, Hefei 230036, China

## Abstract

Plant ribosomes contain four specialized ribonucleic acids, the 5S, 5.8S, 18S, and 25S ribosomal RNAs (rRNAs). Maturation of the latter three rRNAs requires cooperative processing of a single transcript by several endonucleases and exonucleases at specific sites. In maize (*Zea mays*), the exact nucleases and components required for rRNA processing remain poorly understood. Here, we characterized a conserved RNA 3′-terminal phosphate cyclase (RCL)-like protein, RCL1, that functions in 18S rRNA maturation. *RCL1* is highly expressed in the embryo and endosperm during early seed development. Loss of *RCL1* function resulted in lethality due to aborted embryo cell differentiation. We also observed pleiotropic defects in the *rcl1* endosperm, including abnormal basal transfer cell layer growth and aleurone cell identity, and reduced storage reserve accumulation. The *rcl1* seeds had lower levels of mature 18S rRNA and the related precursors were altered in abundance compared with wild type. Analysis of transcript levels and protein accumulation in *rcl1* revealed that the observed lower levels of zein and starch synthesis enzymes mainly resulted from effects at the transcriptional and translational levels, respectively. These results demonstrate that RCL1-mediated 18S pre-rRNA processing is essential for ribosome function and messenger RNA translation during maize seed development.

## Teaser

Loss of RCL1 results in defective 18S pre-rRNA processing and decreased translation efficiency, leading to abnormal embryo differentiation and storage reserve synthesis during maize kernel development.

IN A NUTSHELL **Background:** Every living cell requires ribosomes to produce proteins from amino acids via a process called protein synthesis or translation. A ribosome is a massive, complex structure composed of RNA (rRNA) and many proteins. It is composed of two subunits—the smaller (40S) and the larger (60S). The messenger ribonucleic acid (mRNA) binds and is decoded in the 40S subunit and the amino acids are added in the 60S subunit. In plants, the 40S small subunit is composed of various ribosomal proteins and 18S rRNA, while the large 60S subunit contains 5S, 5.8S, and 25S rRNAs, in addition to ribosomal proteins. The 5.8S, 18S, and 25S rRNAs in maize are transcribed as a single precursor transcript. This precursor rRNA (pre-rRNA) transcript is processed by numerous factors through various pathways.

**Question:** What are the crucial factors that are involved in the processing of 18S rRNA? What is the outcome if abnormal 18S rRNA maturation occurs during maize kernel development?

**Findings:** We isolated a maize seed mutant that exhibits arrested embryo differentiation, opaque and shrunken endosperm, and reduced storage protein and starch. We cloned the causal gene and confirmed that it encodes a conserved nucleolar RNA 3′-terminal phosphate cyclase-like protein (RCL1). We showed that RCL1 participated in the processing of 18S pre-rRNA. Consistent with the lower level of 18S rRNA, we found that the amounts of 40S subunit, 80S ribosome and polysomes were reduced in the *rcl1* mutant compared with the wild type. Furthermore, we showed that the translation efficiency of key transcription factors for storage protein synthesis and key enzymes for starch synthesis was affected. Therefore, we discovered an essential protein for 18S pre-rRNA processing in maize. The absence of this enzyme disrupted ribosome biogenesis and protein translation during maize kernel development.

**Next steps:** To better elucidate the underlying molecular mechanism of RCL1 in 18S rRNA processing, we will identify the RCL1 associated proteins and other components that are required for 18S rRNA maturation in maize.

## Introduction

The dry seeds of maize (*Zea mays*) contain three major parts: the pericarp, which originates from maternal tissue, and the diploid embryo and triploid endosperm, which originate from double fertilization of the haploid egg cell, and the dikaryotic central cell, respectively (Sabelli and Larkins, 2009; Armistead and Triggs-Raine, 2014). The embryo in the mature seed has developed into a miniature plant including five to six leaf primordia and a primary root meristem, which is essential for germination. In contrast to the small embryo, the maize endosperm occupies a large volume of the seed, makes up a large proportion of the seed dry weight, and stores most of the proteins and carbohydrates required for early seedling development and used for human food and animal feed (Fontanet and Vicient, 2008). The endosperm provides nutrition during the early days of embryo germination (Lopes and Larkins, 1993).

In maize embryo development, the fertilized diploid zygote first undergoes an asymmetric division at 0–10 days after pollination (DAP). Next, meristematic cells differentiate into the shoot apical meristem (SAM) and root primordium, which develop into shoot/root apex, mesocotyl, and cotyledon. Initial endosperm development involves formation of the coenocyte in the first 2 DAP. From 3 to 6 DAP, endosperm cells began to cellularize and differentiate, producing four distinct cell types: starchy endosperm, aleurone, basal endosperm transfer layer (BETL), and embryo-surrounding region. Then, during mitotic proliferation, endosperm cells gradually switch to endoreduplication and synthesize storage proteins and starch (Leroux et al., 2014; Zhan et al., 2015). Successful differentiation of endosperm and embryo cells is essential to produce storage proteins and carbohydrates, and for the life cycle transition. Because of the vigorous cell differentiation activity and storage reserve biosynthesis, seed development requires vast amounts of ribosomes.

The ribosome is a conserved protein synthesis machine that functions by association with a messenger ribonucleic acids (mRNAs) and decodes the information into amino acid chains. The biogenesis of plant ribosomes, including rDNA transcription, precursor rRNA (pre-rRNA) processing and modification, and assembly of ribosome proteins, involves hundreds of ribosome biogenesis factors (Tomecki et al., 2017; Saez-Vasquez and Delseny, 2019). Although ribosome structure is similar across eukaryotes, the exact composition varies depending on the organism (Doudna and Rath, 2002; Nierhaus, 2009; Wilson and Doudna Cate, 2012). In plants, mature ribosomes (80S) contain more than 80 ribosome proteins, a large subunit (60S) and a small subunit (40S) containing four different ribosomal RNAs (rRNAs: 18S, 5.8S, 25S, and 5S) in total. The 40S small subunit contains only the 18S rRNA and the remaining three rRNAs are targeted to the large subunit (Weis et al., 2015; Saez-Vasquez and Delseny, 2019). Therefore, the biogenesis of the 18S rRNA is crucial for mature 80S ribosome assembly.

Ribosome biogenesis begins with transcription of the tandemly repeated 45S rDNA by RNA polymerase I in the nucleolus, to produce the 45S pre-rRNA, a polycistronic transcript. The 45S pre-rRNA, which is cleaved to generate the 35S pre-rRNA intermediate, contains the 18S, 5.8S, and 25S pre-rRNAs separated by two internal transcribed spacers (ITS1 and ITS2), and flanked by two external transcribed spacers (5′-ETS and 3′-ETS) (Boisvert et al., 2007; Saez-Vasquez and Delseny, 2019). Removal of the flanking ETS and ITS is mediated by numerous ribonucleoprotein factors (Fernandez-Pevida et al., 2015; Henras et al., 2015; Tomecki et al., 2017; Saez-Vasquez and Delseny, 2019).

The processing of 35S pre-rRNA in yeast (*Saccharomyces cerevisiae*) has been intensively characterized (Kos and Tollervey, 2010; Henras et al., 2015). Recently, the precise processing sites of 35S pre-rRNA in rice (*Oryza sativa*) and maize have also been mapped (Hang et al., 2018; Liu et al., 2020). The 35S rRNA is cleaved by two alternative pathways, named the major ITS1-first pathway and the minor 5′ -ETS-first pathway according to which site is cleaved first (Supplemental Figure S1). In the ITS1-first pathway, cleavage at the A_3_ site separates the large and small subunit rRNA components, generating A3-B2 and P-A3 pre-rRNA intermediates. Then processing of P-A3 at the P′, A_1_, A_2_, and D sites forms the 18S mature rRNA (Supplemental Figure S1). In the 5′ ETS-first pathway, which is the primary pathway in yeast, cleavage at the P′ site (A_0_ in yeast) produces the 33S pre-rRNA (P′ -B2), which is then processed rapidly at sites A_1_ and A_2_ to generate the 32S (A1-B2) and 20S pre-rRNA (18S-A2), respectively. Further processing at the D site generates the mature 18S rRNA (Supplemental Figure S1). Both pathways can yield the 18S rRNA.

The sites and the order of endonuclease and exonuclease processing that remove the 5′ ETS and ITS1 are well defined in yeast and humans (Tomecki et al., 2017; Cerezo et al., 2019). To date, at least three nucleases in yeast were shown to participate directly in 18S pre-rRNA cleavage. The PilT N-terminal (PIN) domain endonuclease Utp24 cleaves 18S pre-rRNA at A_1_ and A_2_ sites in yeast simultaneously, and human UTP24 also functions analogously (Bleichert et al., 2006; Wells et al., 2016; An et al., 2018). Another PIN family endoribonuclease, Nob1, digests the 18S pre-rRNA at site D, leading to the production of mature 18S rRNA (Fatica et al., 2003; Lamanna and Karbstein, 2009; Ameismeier et al., 2020).

RNA 3′-terminal phosphate cyclase-like protein, Rcl1p, is a conserved nucleolar nuclease that cleaves pre-rRNA mimics at site A_2_, and mutations in *Rcl1p* disrupt 18S rRNA processing and 40S subunit assembly in yeast (Billy et al., 2000; Horn et al., 2011). Loss of function of *Nob1*, *Rcl1p* and *Utp24* in yeast and the *Utp24* ortholog in *Arabidopsis thaliana* are lethal, indicating that 18S maturation is essential for the cellular processes (Fatica et al., 2003; Bleichert et al., 2006; Horn et al., 2011; Missbach et al., 2013; Wells et al., 2016). Although the involvement of 18S rRNA in plant development has been established in Arabidopsis, the function of RCL family proteins in other plants is unknown, and its role in endosperm development and filling remains to be defined.

In this study, we report the identification of the maize *rcl1* mutant, which exhibits arrested embryo differentiation, opaque endosperm, and shrunken seeds with decreased storage protein and starch content. *RCL1* is expressed in the embryo and endosperm cells during seed development. We found that RCL1 is located in the nucleolus and is required for 18S rRNA processing and ribosome formation. Consequently, the translation of critical genes for storage protein and starch synthesis enzymes is inhibited in the *rcl1* mutants.

## Results

### The *rcl1* mutant exhibits aberrant embryo and endosperm development

**Figure 1:**
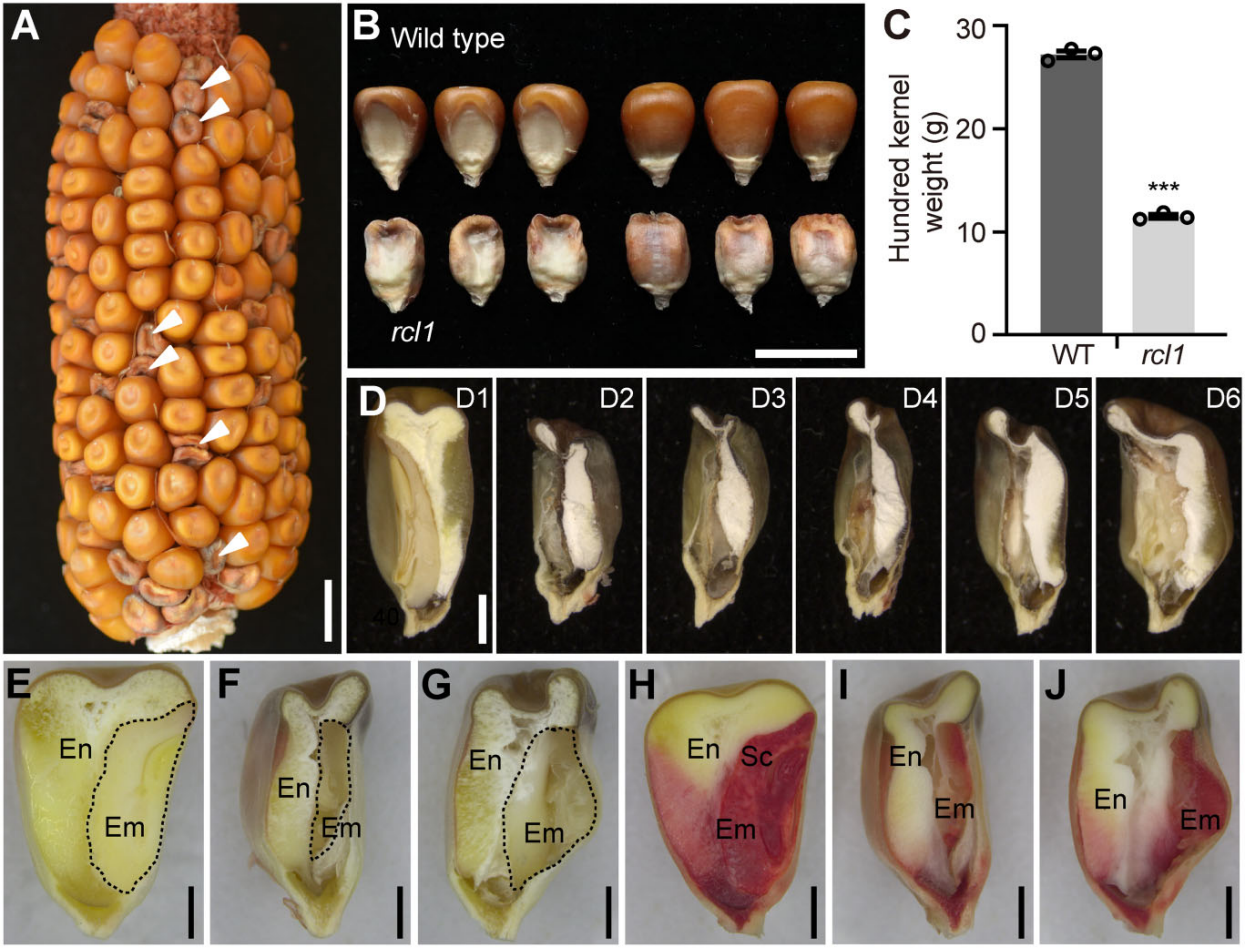
Kernel phenotype of *rcl1*. A, Self-pollinated ear from the heterozygous plant. White arrowheads indicate homozygous *rcl1* kernels. Bar = 1 cm. B, Kernel phenotypes of the WT and *rcl1* mature seeds. Bar = 1 cm. C, Comparison of hundred kernel weight of WT and *rcl1.* Seeds weight was measured from three self-pollinated *rcl1*/+ plants. Error bar represents ±SEM of the three ears (*n* = 50 for each ear). ^***^P < 0.001, Student’s *t* test. D, Longitudinal hand dissections of mature kernels of WT (D1) and *rcl1* (D2–D6). Bars = 2 mm. E–G, Longitudinal hand dissections of 30 DAP kernels of WT (E) and *rcl1* (F and G). Areas with dash lines showed the embryos. Bars = 2 mm. H–J, Seeds of WT (H) and *rcl1* (I and J) stained with 2,3,5-triphenyltetrazolium chloride. The embryo tissues showed red color due to normal live-cell permeability. Bars = 2 mm. Em, embryo; En, endosperm; Sc, scutellum.

While propagating active *Mutator* (*Mu*) lines from the Maize Genetics Cooperation stock center (McCarty et al., 2005), three self-pollinated ears segregating mutated kernels were unexpectedly discovered from the UFMu-02204 line and were designated *rcl1* (Figure 1A). Because homozygous *rcl1* seeds did not germinate (Supplemental Figure S2A), we maintained this mutant as *rcl1/+* heterozygotes. The self-pollinated ears of the *rcl1/+* plants segregated mutant and normal kernels in a 1 : 3 ratio (166 : 489, *P* > 0.95). The kernel phenotype and the segregation ratio were stable after several generations of propagation, indicating that a recessive mutation in a nuclear gene causes the *rcl1* phenotype.

In contrast to the wild-type (WT), the *rcl1* kernels were generally shrunken, with rough endosperm, sunken embryo, and significantly reduced kernel weight (Figure 1, B and C). Further comparison and statistical analysis revealed that kernel length, width, and thickness of *rcl1* mutant were all reduced (Supplemental Figure S2, B–E). The WT kernel at the mature stage contains an embryo, hard (vitreous) endosperm, and soft (opaque) endosperm (Figure 1D, D1). Longitudinal hand sections of mature *rcl1* kernels showed an aborted embryo and opaque endosperm (Figure 1D, D2–D6). The endosperm in *rcl1* mutants was generally opaque and smaller compared with that in WT, but contained a bigger interspace at the bottom (Figure 1D). The kernel mutant phenotype could be distinguished from the WT siblings as early as 12 DAP, since kernels in *rcl1* at this stage were watery and smaller than WT (Supplemental Figure S2F).

Embryonic structures of *rcl1* seeds were more variable in size than WT, and were distorted or even absent or degraded. Dissection of *rcl1* kernels at 30 DAP revealed aberrant embryo structures (Figure 1, E–G). Live embryo cells exhibited membrane permeability and could be stained by 2,3,5-triphenyltetrazolium chloride (Foster et al., 2012). After staining, 30 DAP WT kernels showed intensely stained embryos with differentiated scutellum and leaf primordium while *rcl1* seeds contained less stained endosperm and embryo cells with indistinguishable embryonic identity (Figure 1, H–J). These observations showed that both endosperm and embryo development were deeply affected in the *rcl1* kernels.

### The *rcl1* mutant shows multiple cell differentiation defects

**Figure 2:**
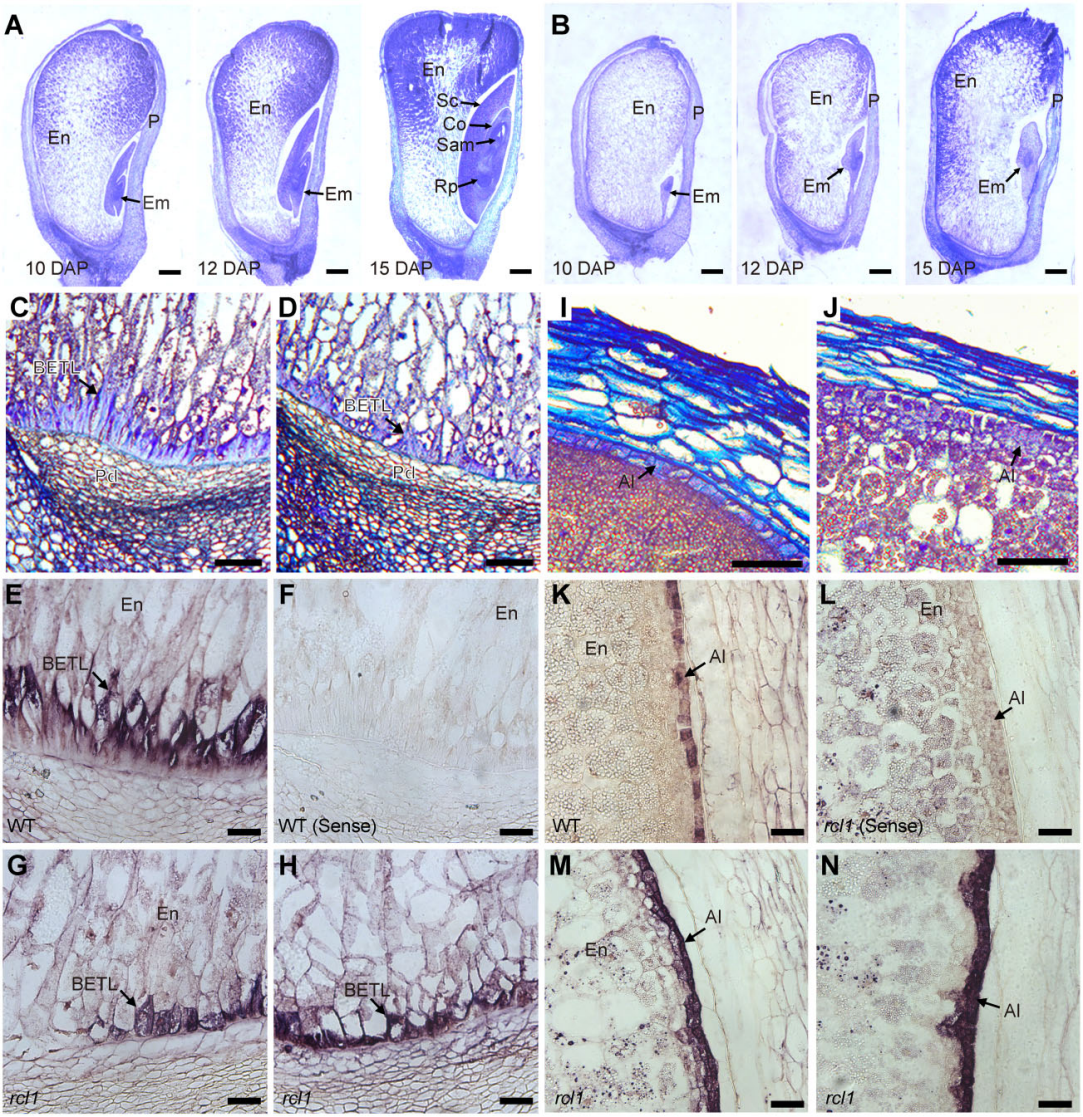
Light microscopy observations and in-situ hybridization analysis of morphology defects in *rcl1*. A and B, Light microscopy observations of paraffin-embedded developing kernels of WT (A) and *rcl1* (B). Bars = 1 mm. C and D, Light microscopy observations of BETL in WT (C) and *rcl1* (D) mutant. Bars = 100 μm. E–H, RNA in situ hybridization using BETL-specific *Miniature1* probe in WT (E) and *rcl1* mutant (G) and (H). Hybridized signals are shown in purple in BTEL cells. Sense probe produces no hybridization signal in WT (F). Bars = 200 μm. I and J, Light microscopy observations of aleurone in WT (I) and *rcl1* (J) mutant. Bars = 100 μm. K–N, RNA in situ hybridization using aleurone-specific *AL9* probe in WT (K) and *rcl1* mutant (M) and (N). Sense probe produces no hybridization signal in *rcl1* (L). Bars = 200 μm. Al, aleurone; BETL; basal endosperm transfer layer; Co, Coleoptile; Em, embryo; En, endosperm; Rp, root primordium; P, pericarp; Pd, pedicel; Sc, scutellum; Sam, shoot apical meristem.

To examine the developmental defects in the *rcl1* mutant, we made paraffin-embedded sections of WT and *rcl1* kernels from the same segregating ear and observed the sections by light microscopy. At 10 DAP, WT embryos began to differentiate and form a visible scutellum. Typical cell patterning with leaf primordia, SAM, and primary root primordium was observed at 15 DAP in WT embryos (Figure 2A). Consistent with the above observations, the *rcl1* embryos were much smaller than those in the WT. Moreover, the *rcl1* embryo cells remained homogeneous and did not form any differentiated meristem and scutellum by 15 DAP (Figure 2B).

BETL in the lower portion of the endosperm showed specialized cell wall ingrowths and is essential for the uptake of solutes for seed filling and development (Gomez et al., 2002). We also found that BETL morphology in *rcl1* is impaired. The BETL was characterized in WT kernels as 1–2 layers of relatively large, elongated cells with both reticulate and flange cell wall growths (Figure 2C). By contrast, the *rcl1* BETL developed less-elongated cells and had very few cell wall ingrowths (Figure 2D). RNA in situ hybridization using the BETL specific *Miniaturel1* gene (Carlson et al., 2000) further revealed aberrant and discontinuous BETL morphology with less cell wall ingrowth in *rcl1*, compared with that in normal siblings (Figure 2, E–H).

The aleurone is a single cell layer located at the outermost boundary of the endosperm (Leroux et al., 2014). Aleurone was distinguishable in the 15 DAP WT kernel based on the boxy shape and dense cytoplasm of its cells (Figure 2I). In *rcl1*, aleurone cells contained more than one layer with irregular structure (Figure 2J). We also compared the aleurone cell pattern by in situ hybridization with the *Aleurone 9* (*AL9*) RNA (Zhan et al., 2015). WT endosperm produced a single layer of aleurone (Figure 2K). By contrast, the significantly increased signal in *rcl1* endosperm revealed an additional aleurone layer in certain areas and altered cell properties (Figure 2, M and N). Collectively, these results showed that RCL1 is required for both embryogenesis and endosperm cell development during kernel development.

### The *rcl1* mutants show endosperm filling defects

**Figure 3:**
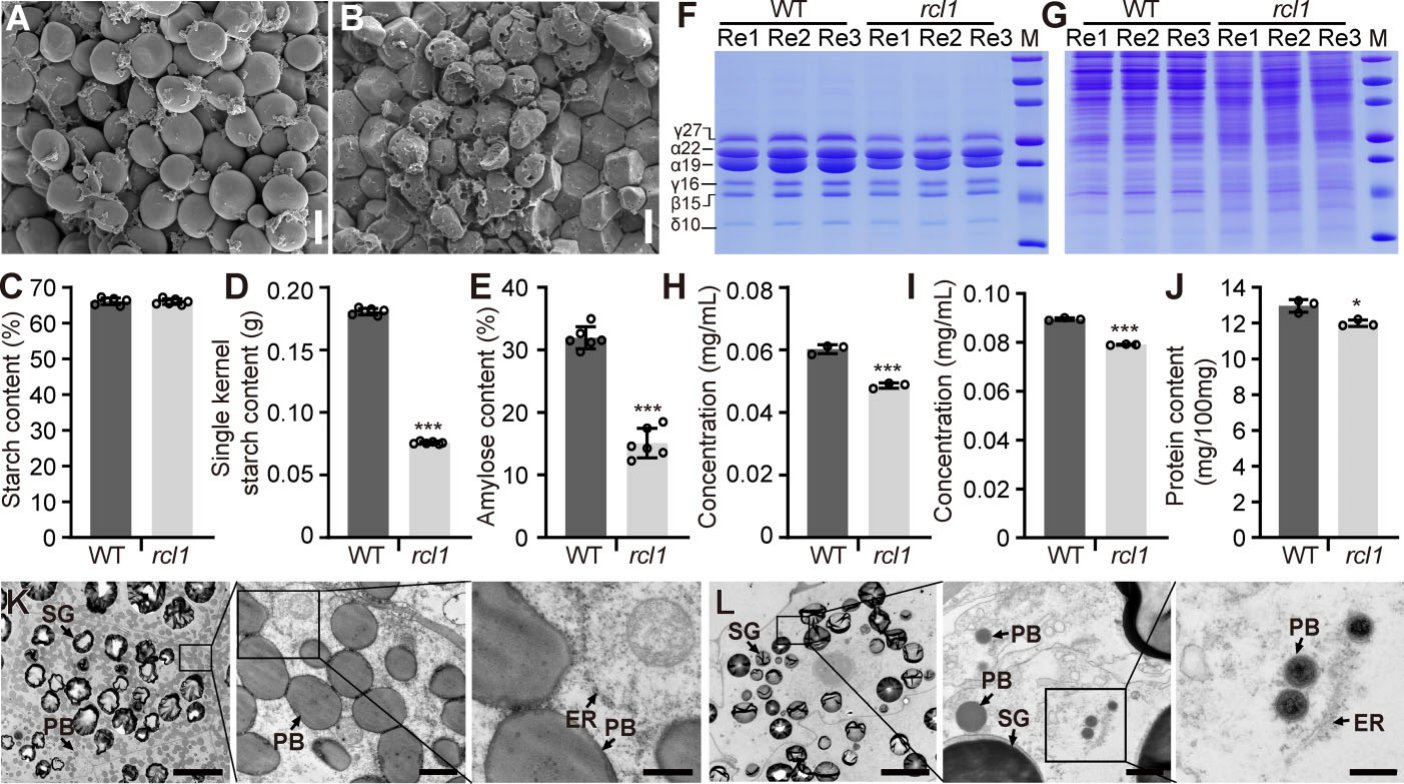
Analysis of starch and protein in the WT and *rcl1*. A and B, Scanning electron microscopy observation of starch granules from central areas of WT (A) and *rcl1* (B) mature seeds. Bars = 10 μm. C–E, Measurement of total starch (C), single kernel starch (D), and amylose (E) in WT and *rcl1* dry seeds. Six biological replicated samples from different ears were prepared for analysis. F and G, SDS-PAGE analysis of zein (F) and non-zein (G) in mature seeds of WT and *rcl1*. Protein markers from top to bottom are 75, 50, 37, 25, 20, 15, and10 kDa. Re1, Re2, and Re3 mean three replicated proteins from different ears. H and I, Quantification of zein (H) and non-zein protein (I) concentration as shown in (F) and (G) using the BCA method. J, Dumas method quantification of total protein contents from three different ears of WT and *rcl1* mature seeds. K and L, Transmission electron microscopy observation of 15 DAP endosperm in WT (K) and *rcl1* (L). Boxed areas are enlarged in turn. Bars = 10 μm (left), 1 μm (middle), and 0.5 μm (right). ER, endoplasmic reticulum; PB, protein body; SG, starch granule. Error bars represent the ±SD. ^*^P < 0.05, ^***^P < 0.001, Student’s *t* test.

As mature *rcl1* seeds are shrunken and much lighter than WT seeds, we next compared their primary storage material, including starch and proteins. Scanning electron microscopy of mature kernels revealed that the WT starch granules showed globular morphology and accumulated some matrix (Figure 3A). By contrast, in *rcl1*, most starch granules were pitted with an irregular shape and were occasionally cracked or collapsed (Figure 3B). Although the total starch content based on dry endosperm flour was not significantly different (Figure 3C), the *rcl1* starch content per seed (0.07 g) is reduced compared with that of the WT (0.18 g) due to substantially lower kernel weight (Figure 3, D and C). Amylose content is drastically reduced in *rcl1* endosperm (Figure 3E), so the composition of starch is changed.

Zein proteins in the seeds are assembled into protein bodies that fill the spaces among starch granules and generate the vitreous endosperm texture (ZHang et al., 2018; Li and Song, 2020). Based on amino acid sequence and structure, zein proteins can be categorized into four classes: α- (19 and 22 kDa), β- (15 kDa), γ- (50, 27, and 16 kDa), and δ- (18 and 10 kDa). The opaque endosperm phenotype in *rcl1* led us to test whether zein accumulation is affected. Zein proteins from developing *rcl1* and WT kernels were extracted and then analyzed by SDS-PAGE. The synthesis of zeins in WT could be detected at 12 DAP; however, *rcl1* zein synthesis was severely delayed and was not observed until 20 DAP (Supplemental Figure S3A). Moreover, at the mature stage, zein levels were also reduced in the *rcl1* endosperm compared with those of the WT (Figure 3F), suggesting a general effect of *rcl1* on zein synthesis. SDS-PAGE analysis showed that the non-zein protein levels in *rcl1* were also lower relative to WT (Figure 3G and Supplemental Figure S3B). We next measured the protein concentration from mature kernels and found that *rcl1* zein and non-zein protein concentrations were lower than the WT (Figure 3, H and I). We further compared the total protein content using the Dumas combustion method (Maria, 2020). Consistently, *rcl1* seeds showed 9.2% less protein than WT, based on dry seed flour (Figure 3J).

Consistent with the delayed zein protein synthesis during kernel development, transmission electron microscopy observation of the endosperm at 20 DAP showed that the number and size of protein bodies in *rcl1* were strikingly reduced and that the appearance of the endoplasmic reticulum (ER) was slightly irregular, in contrast to that in the WT (Figure 3, K and L). These analyses suggest that the *rcl1* mutation has extensive effects on storage reserve deposition.

### Positional cloning and genetic confirmation of *RCL1*

**Figure 4:**
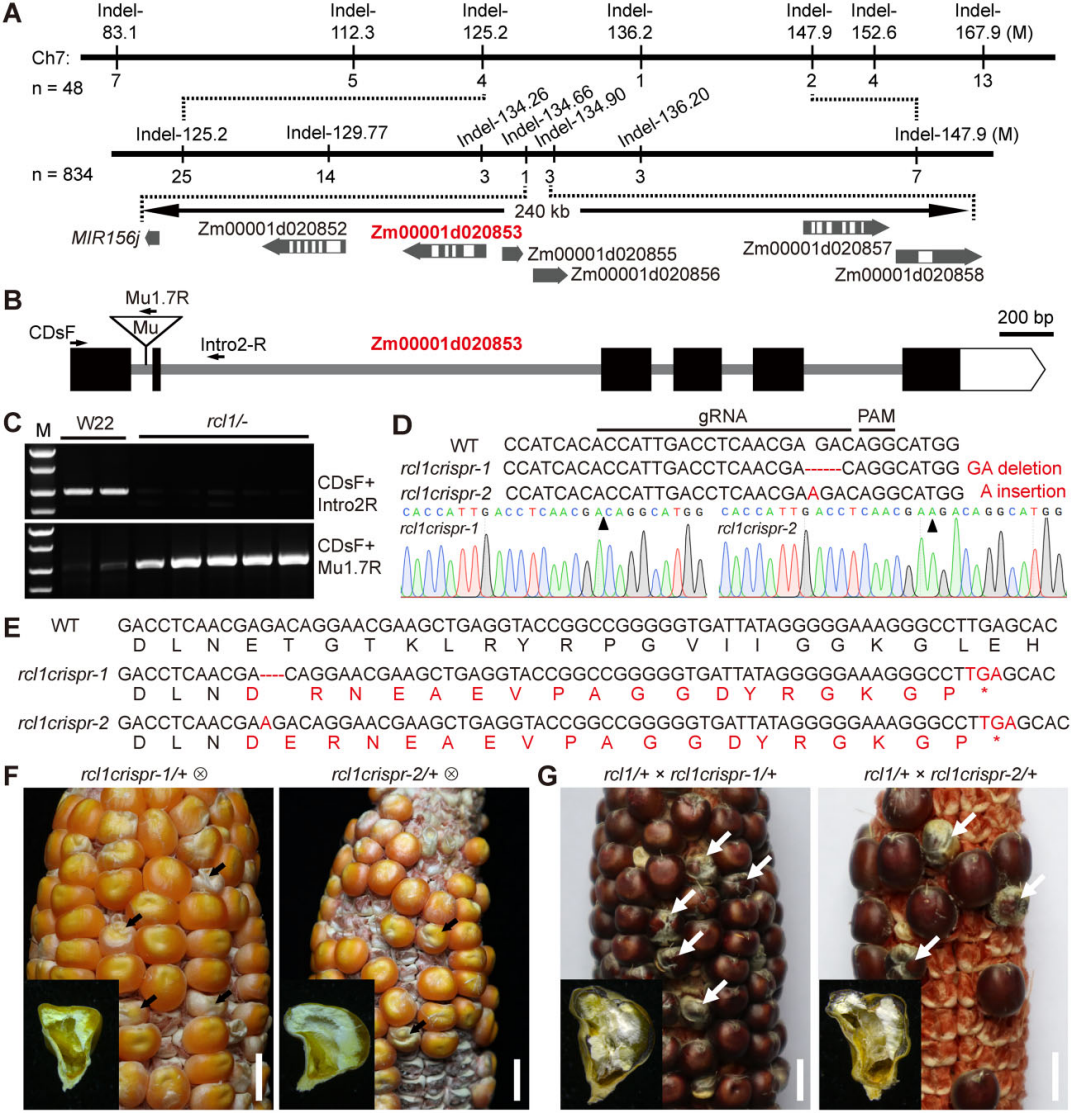
Map-based cloning and allelic confirmation *RCL1*. A, Fine-mapping processes of *RCL1* using an F_2_ population crossed between B73 and *rcl1*/+. The numbers under each vertical bar represent the amounts of recombinants identified by the corresponding indel marker on the top. The gene ID in red indicates the casual gene. B, Schematic diagram showing the *RCL1* gene structure. The black boxes and gray lines indicate exons and introns, respectively. The triangle represents *Mu1.7* insertion in the first intron. C, Amplification of the *Mu1.7* insertion in homozygous *rcl1* and W22 inbred line using primer pairs indicated in (B). DNA marker from top to bottom is 2.0, 1.5, 1.0, and 0.75 kb. D, Illustration of genome-edited *rcl1* alleles by CRISPR/Cas9. The gRNA and protospacer adjacent motif (PAM) sequence are indicated. The resulted *rcl1crispr-1* and *rcl1crispr-2* lines contain 2-bp (GA) deletion and 1-bp (A) insertion, respectively (indicated by arrowheads). E, Nucleic acid and deduced amino acid sequences from WT, *rcl1crispr-1*, and *rcl1crispr-2*. The red font highlights the mutated amino acid sequences. Both transgenic lines lead to frameshift and premature stop codons. F, Self-pollinated ears of *rcl1crispr-1*/+ (left) and *rcl1crispr-2*/+ (right). The mutant seeds are indicated by arrows. Bars = 1 cm. G, Ears of a *rcl1/+* ear crossed by pollen from *rcl1crispr-1*/+ (left) and *rcl1crispr-2*/+ (right) plants. Arrows indicate the mutant seeds. Bars = 1 cm.

Homozygous *rcl1* is lethal; therefore, we crossed *rcl1/+* heterozygous pollen to B73 ears and created an F_2_ population from self-pollinated F_1_. By screening whole-genome Indel (insertion or deletion) markers using 48 mutant individuals of F_2_, the *rcl1* locus was mapped to a 22.7-Mb region of chromosome 7 (Figure 4A). After fine-mapping with a total of 834 individuals, the causal gene was narrowed to a 240-kb interval between the chromosomal position of 134.66 and 134.90 Mb. Based on the maize genome database data (Jiao et al., 2017), six protein-coding genes and a microRNA gene (*MIR156j*) were annotated in this region. Published RNA-seq data (Chen et al., 2014; Li et al., 2014) showed four of the seven genes expressed in seeds from 4 to 36 DAP (Supplemental Figure S4A). Among those four genes, Zm00001d020853 is expressed widely at all developmental stages and with the highest level (Supplemental Figure S4A). We selected this gene as the candidate for *rcl1* and subjected it to further analysis.

Since the *rcl1* mutant was created by *Mu* transposon insertion (McCarty et al., 2005), we combined different *Mu*-specific primers with Zm00001d020853 genome sequences as PCR primer pairs. A particular PCR product was obtained when the Tir8.3 primer was used. Sequencing the product revealed a *Mu1.7* transposon insertion in the first intron of Zm00001d020853 + 288-bp from the start codon (Figure 4B). Co-segregation analysis by genotyping the progeny of *rcl1/+* plants showed that the *rcl1* mutants were homozygous for this insertion (Figure 4C). In contrast, the normal seeds were either heterozygous or lacked the *Mu* insertion, indicating a tight linkage between the *Mu1.7* insertion and the *rcl1* phenotype (Supplemental Figure S4B).

To validate that Zm00001d020853 is the causal gene for *rcl1*, we performed a site-specific mutation experiment using the clustered regularly interspaced short palindromic repeats (CRISPR)/Cas9 system in the C01 background (Li et al., 2017). The guide RNA (gRNA) spacer sequence targeting the first exon (184–203 bp) was chosen for vector construction (Figure 4D). After embryo transformation and selection, we recovered two independent transgenic lines harboring heterozygous frame-shift mutations caused by a two-base deletion (-GA) or one-base insertion (+A); these were designated *rcl1crispr-1* and *rcl1crispr-2*, respectively (Figure 4D). Both of the lines generate premature stop codons in the new reading frame and, therefore, are predicted to encode truncated proteins (Figure 4E). Self-pollinated ears from *rcl1crispr-1/+* and *rcl1crispr-2/+* plants segregated normal and mutant seeds (Figure 4F). We next performed an allelism test between *rcl1/+* and the above two edited lines. When the pollen of *rcl1crispr-1/+* and *rcl1crispr-2/+* tassels was used to pollinate *rcl1/+* ears, the hybrid ears from both lines segregated normal and mutant seeds with a similar phenotype to the *rcl1* homozygous mutant (Figure 4G).

To further confirm Zm00001d020853 as the causal locus for *rcl1*, we next performed genetic complementation using FLAG-tagged Zm00001d020853 coding sequence driven by the *Ubiquitin* promoter (Supplemental Figure S5A). Six transgenic events were obtained after maize transformation (Supplemental Figure S5B). Because the *rcl1* mutant is lethal, pollen from transgenic lines was used to pollinate *rcl1/+* ears. We then self-pollinated the F_1_ plants and germinated the resulting F_2_ seeds to isolate complemented seedlings that were homozygous for the *Mu1.7* insertion in *RCL1*. Among the F_2_ seedlings from two independent crossed lines, we obtained at least ten complemented seedlings that were homozygous for the *Mu1.7* insertion and carried the transgenic fragment (Supplemental Figure S5, C and D). Immunoblotting showed that FLAG-tagged RCL1 is expressed in the complemented kernels (Supplemental Figure S5E), suggesting that this fusion protein can complement the *rcl1* lethal phenotype (Supplemental Figure S5F). These genetic data together demonstrated that Zm00001d020853 is the gene responsible for the *rcl1* mutant phenotype.

### *RCL1* is constitutively expressed in maize tissues

**Figure 5:**
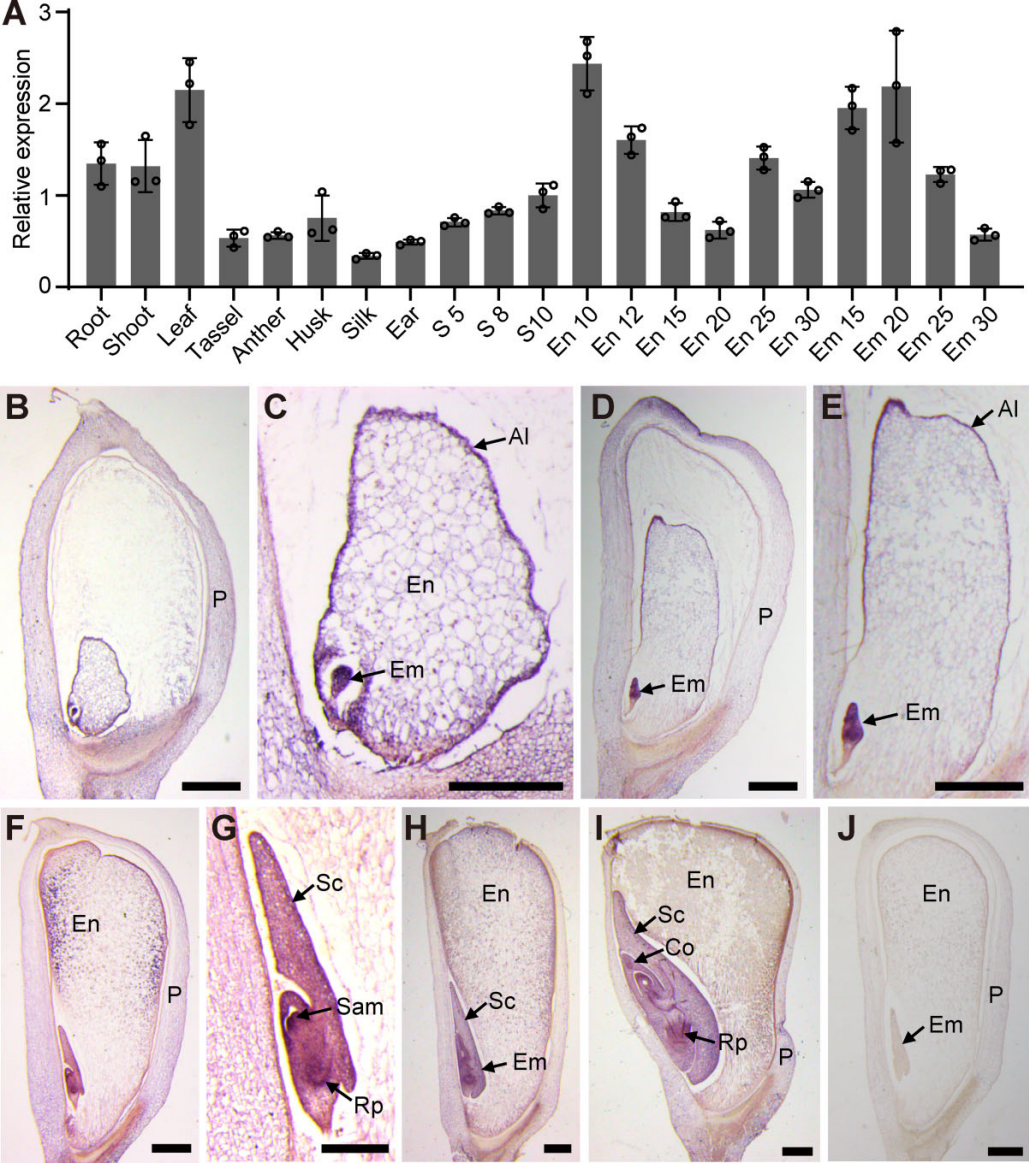
The expression pattern of *RCL1* gene. A, RT-qPCR analysis of *RCL1* expression in different B73 tissues as indicated. The expression levels are normalized to the maize *Ubi* gene. The numbers after each tissue indicate the DAP. Error bras represent ±sd from three technical replicates. Experiments were repeated three times using biological samples from different ears with similar results. B–I, RNA in situ hybridization of *RCL1* probe in B73 kernels at 5 DAP (B) and (C), 8 DAP (D) and (E), 10 DAP (F) and (G), 15 DAP (H) and 20 DAP (I). Hybridized signals are shown in purple. Bars = 2 mm. J, 10 DAP B73 kernel hybridized with sense probe. Bar = 2 mm. Al, aleurone; Co, coleoptile; Em, embryo; En, endosperm; S, seed; Sam, shoot apical meristem; P, pericarp.

According to the RNA-seq gene atlas of maize, the *ZmRCL1* transcript can be detected in all tissues (Chen et al., 2014; Walley et al., 2016). The results of reverse transcription quantitative PCR (RT-qPCR) showed that *ZmRCL1* is widely expressed in maize tissues including root, leaf, stem, tassel, anther, husk, ear, and seed at 10 DAP. The expression of *RCL1* in leaf, root, and shoot is higher than that in the other tissues analyzed (Figure 5A). Due to the apparent kernel phenotype of *rcl1*, we also investigated the *RCL1* expression pattern during seed development. We used the whole seeds at 5–8 DAP and the embryos or endosperms at later stages for RNA extraction. RT-qPCR detected *RCL1* expression in all tissues at different stages, with a slightly higher expression level in the 10 DAP endosperm and the embryo at 20 DAP (Figure 5A).

In situ hybridization analysis was performed to examine the temporal and spatial distribution of *RCL1* expression in the kernel. The results revealed more robust expression of *RCL1* in the embryo cells and aleurone of the endosperm than in the inner endosperm and maternal tissues at 5 and 8 DAP (Figure 5B–E). In the 10 DAP seed, *RCL1* signal can be observed in the aleurone, peripheral starchy endosperm, SAM, and primary root primordium (Figure 5, F and G). Later, at 12 and 20 DAP, *RCL1* mRNA was present more in the embryo than in other cells (Figure 5, H and I). No hybridization signal was detected with an *RCL1* probe in the inner starchy endosperm or a sense probe (Figure 5, H–J). The high expression of *RCL1* in the embryo and aleurone is consistent with the developmental defects observed in *rcl1* seeds.

### *RCL1* encodes a conserved nucleolar RCL protein

**Figure 6:**
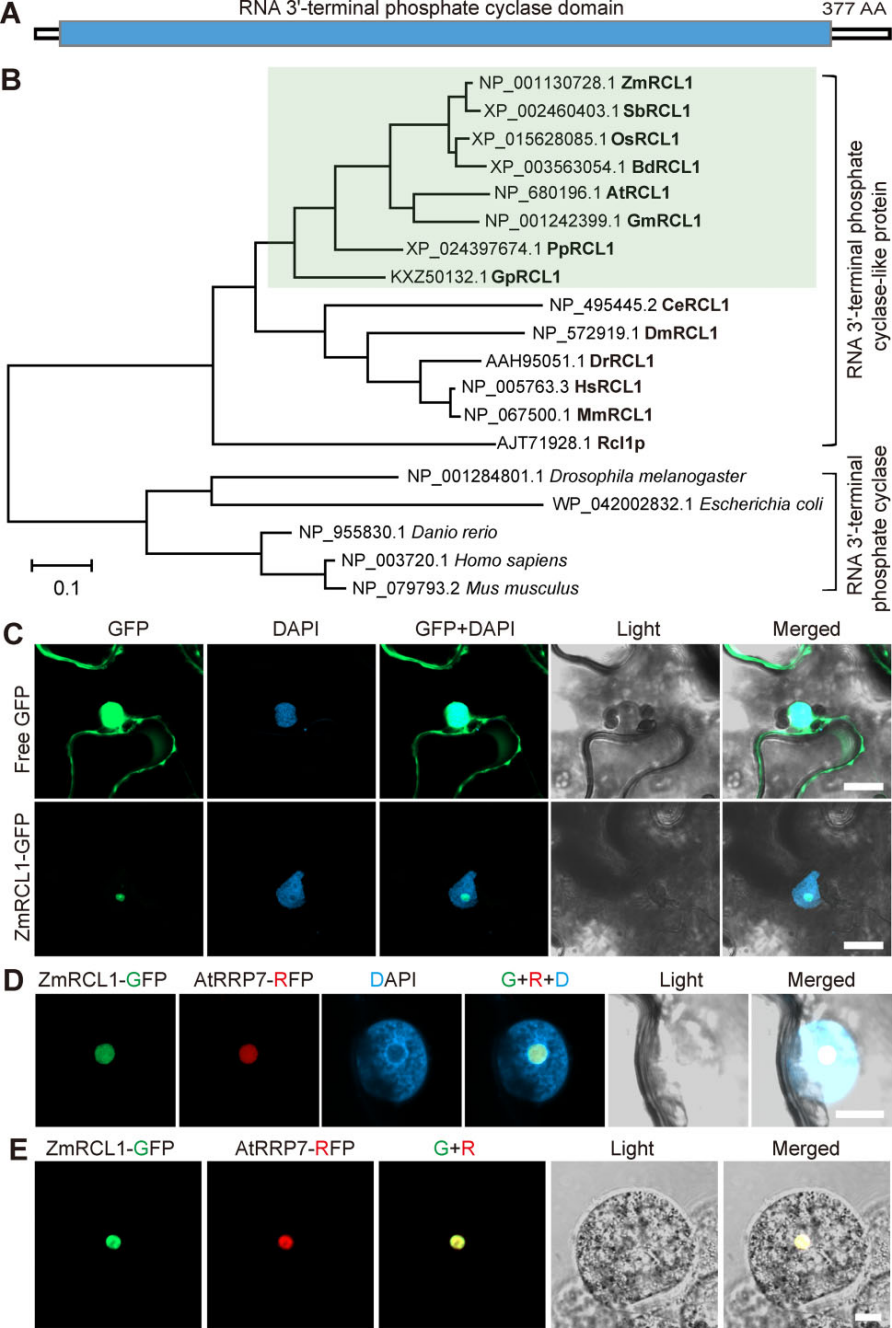
Domain, phylogenetic, and subcellular localization analysis of RCL1. A, Diagram of the RCL1 protein structure. The blue box indicates conserved RNA 3′-terminal phosphate cyclase domain. AA, amino acids. B, Phylogenetic tree of putative RNA 3′-terminal phosphate cyclase and RNA 3′-terminal phosphate cyclase-like proteins (RCL) in representative species. The green boxed area indicates RCL proteins in plants. The scale bar represents the number of substitutions per site. ZmRCL1, *Z. mays*; SbRCL1, *Sorghum bicolor*; OsRCL1, *O. sativa*; BdRCL1, *Brachypodium distachyon*; AtRCL1, *A. thaliana*; GmRCL1, *Glycine max*; PpRCL1, *Physcomitrium patens*; GpRCL1, *Gonium pectoral*; CeRCL1, *Caenorhabditis elegans*; DmRCL1, *Drosophila melanogaster*; DrRCL1, *Danio rerio*; HsRCL1, *Homo sapiens*; MmRCL1, *Mus musculus*; Rcl1p, and *Saccharomyces cerevisiae*. C, Confocal images of free GFP and ZmRCL1-GFP fusion protein driven by 35S promoter in *N. benthamiana* leaf. The nucleuses are shown by DAPI staining. Bars = 20 μm. D and E, Co-localization of ZmRCL1-GFP and Arabidopsis RRP7 (AtRRp7-RFP) protein in *N. benthamiana* leaf (D) and maize endosperm protoplast (E). The nuclei are shown by DAPI staining. Bar = 10 μm. In C and D, transient expression experiments were repeated 2 times. The observed leaf number is 5 for free GFP, 5 for ZmRCL1-GFP and 8 for ZmRCL1 and AtRRP7 co-localization. At least six maize endosperm protoplasts were observed with similar results.

In MaizeGDB, the Zm00001d020853 gene was annotated as RCL protein (Jiao et al., 2017). Therefore, Zm00001d020853 was named *ZmRCL1*. To predict RCL1 function, we searched the Pfam database (http://pfam.xfam.org) using the ZmRCL1 protein sequence. The result showed that maize RCL1 protein contains one long RNA 3′-terminal cyclase domain (Figure 6A).

A phylogenetic tree was constructed using RCLs and RTCs of representative species ranging from prokaryotes to eukaryotes. The phylogenetic tree can be divided into two clades, RTCs and RCLs. Interestingly, plants lack the clade of RTC proteins, but RCL proteins are widely distributed in plants. ZmRCL1 was most closely related to the RCL proteins in plants, followed by those of animals and yeast (Figure 6B), indicating that RTCs and RCLs differentiated before the divergence of plants and animals. Among plant RCLs, ZmRCL1 was more closely related to its homologs from monocots than dicots (Supplemental Figure S6) and the high similarity of amino acid sequences indicated conserved roles of plant RCLs.

In yeast, Rcl1p has been characterized as an essential nuclease required for rRNA processing and growth (Billy et al., 2000; Horn et al., 2011). When aligned with Rcl1p, maize RCL1 exhibited 35% identity and 54% similarity (Supplemental Figure S7A). Substitution of the endogenous *Rcl1* promoter with a galactose-inducible promoter has been used for a complementation test in yeast (Horn et al., 2011). However, ZmRCL1 expressed under *Rcl1p* promoter failed to complement yeast growth in media lacking galactose (Supplemental Figure S7B). Like ZmRCL1, at least 11 plant ribosome biogenesis factors (such as MTR4 and SMO4) failed to complement mutants of their yeast orthologs (Lange et al., 2014; Weis et al., 2015, Zhang et al., 2015, Micol-Ponce et al., 2020).

To determine the subcellular localization of ZmRCL1, we fused ZmRCL1 with GFP and transiently expressed the fusion protein in *Nicotiana benthamiana* leaves. As a control, the signal generated by 35S:GFP was detected both in nuclei and the cytoplasm. By contrast, the signal of 35S:ZmRCL1-GFP was restricted to a distinct region within the nucleus that is likely to be the nucleolus (Figure 6C). To further verify ZmRCL1 localization, co-localization analyses were performed in *N.* *benthamiana* and maize endosperm using the Arabidopsis nucleolar protein AtRRP7 (Micol-Ponce et al., 2018). Confocal observation showed that ZmRCL1 co-localized well with AtRRP7 (Figure 6, D and E), indicating that ZmRCL1 is a nucleolar protein.

### Loss function of ZmRCL1 affects 18S rRNA maturation

**Figure 7:**
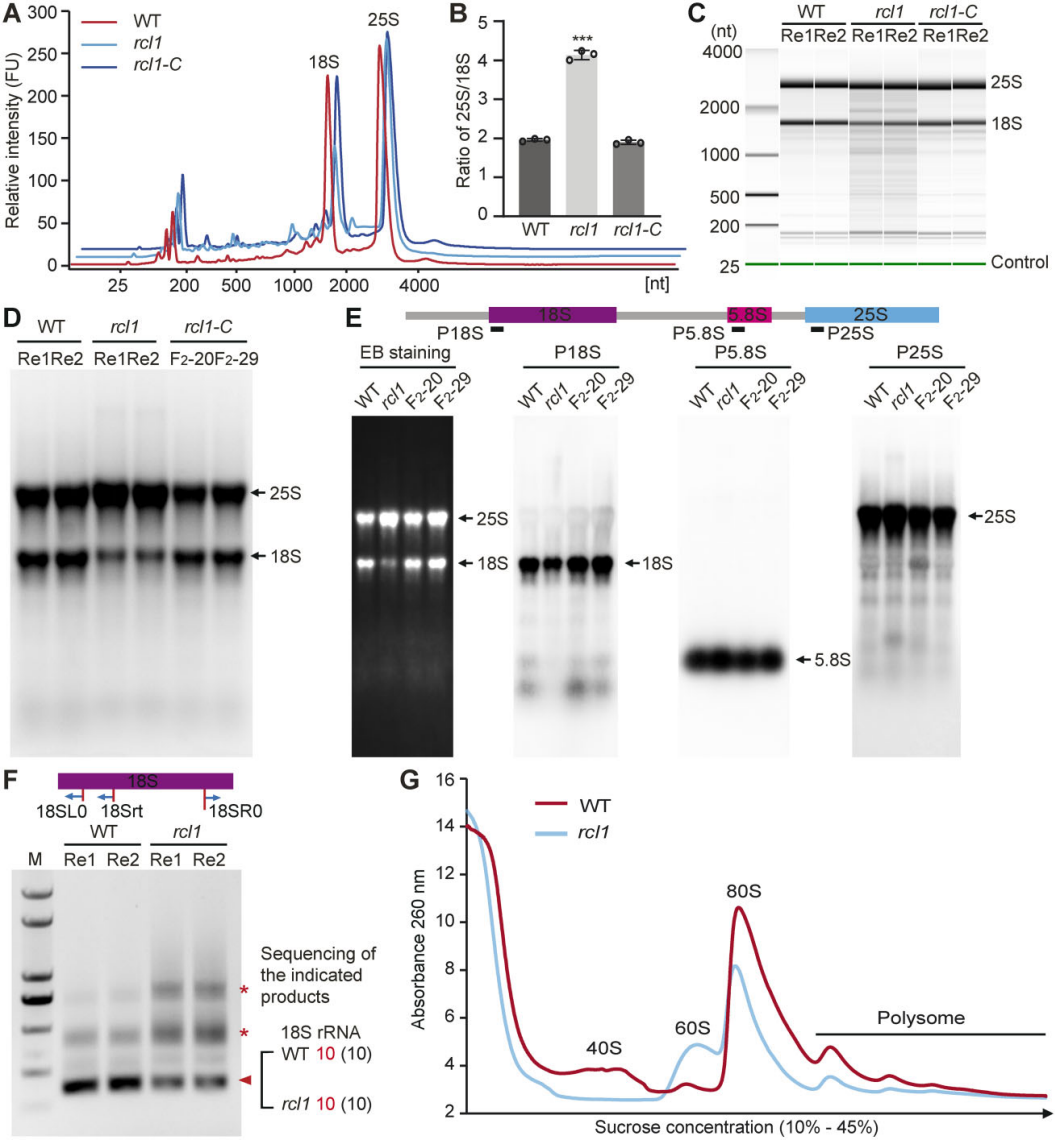
Reduced accumulation of 18S rRNA in *rcl1* mutant. A, Comparison of RNA samples from15 DAP seeds using Agilent RNA 2100 Bioanalyzer. Three RNA samples from different ears of WT, *rcl1*, and *rcl1-C* were analyzed and similar results were obtained. B, Ratio of 25S and 18S rRNA. Error bars represent ±sd from three biological repeated samples. ^***^P < 0.001, Student’s *t* test. C, Simulative gel-like image generated by Agilent RNA 2100 bioanalyzer. Green lines indicate 25-bp internal control. Re1 and Re2 represent the two RNA samples from different ears. D, Denaturing agarose-formaldehyde gel analysis of RNA samples from15 DAP seeds of WT, *rcl1*, and two complemented *rcl1* lines (F_2_-20, F_2_-29). Re1 and Re2 represent two RNA samples from different ears. E, RNA gel blot analysis of the mature rRNA levels. Total RNA was isolated from 15 DAP seeds from WT, *rcl1* and two complemented lines (F_2_-20 and F_2_-29). Equal amount of RNA samples was separated in a denaturing agarose-formaldehyde gel for each probe. An EB staining membrane is shown as a loading control. Positions of rRNA species are indicated on the right. F, Circular RT-PCR analysis using cDNA reverse transcribed with the 18Srt primer. Primers are located in the 5′ and 3′ of mature 18S rRNA. Triangle points to mature 18S rRNA verified by sequencing. Asterisks indicate accumulated bands in *rcl1*. Re1 and Re2 represent two RNA samples from different ears. G, Polysome profiles of WT and *rcl1* mutant. Total seed extractions at 15 DAP were resolved in 10%–45% (w/v) sucrose gradients. The resulted gradient was analyzed by continuous monitoring at A260. The peaks of free 40S and 60S ribosomal subunits, 80S free monosomes and polysomes are indicated. Two repeated samples from different ears were analyzed with similar results. The average value was used to generate the absorbance curve.

The pre-rRNA maturation pathway is conserved and occurs in the nucleolus of eukaryotes. To examine whether 18S rRNA maturation is affected in the absence of nucleolar ZmRCL1, total RNAs from WT, *rcl1* and *rcl1-C* seeds at 15 DAP were extracted and subjected to analysis by the Agilent RNA 2100 Bioanalyzer. The results revealed that the peak of 18S rRNA in *rcl1* was much lower than 25S rRNA compared with that in WT and *rcl1-C* (Figure 7A). The ratio of 25S rRNA to 18S rRNA (25S/18S) is about 2 for high-quality RNA in plants (Fleming et al., 2017). However, the ratio of 25S/18S rRNA in *rcl1* was 4.13, an increase of about two-fold compared with WT (1.95) and *rcl1-C* (1.90) due to the reduced 18S rRNA level (Figure 7B and Supplemental Table S1). Gel mapping from Bioanalyzer or direct ethidium bromide staining showed a decreased level of 18S rRNA, compared with WT and complemented *rcl1-C* kernels (Figure 7, C and D).

To further analyze mature 18S rRNA accumulation, we conducted RNA gel blot analyses using probes targeted to the mature rRNA forms, which demonstrated that *rcl1* mutants had decreased steady-state levels of 18S rRNA but the levels of 5.8S and 25S rRNA were similar to WT (Figure 7E). Next, circular RT-PCR amplification (Figure 7F) showed that the band for the intact mature18S rRNA in RNA from *rcl1* kernels was consistently much lower in intensity compared with WT (Supplemental Figure S8A and Figure 7E). These data suggested that RCL1 was indeed required for 18S rRNA accumulation, not for 45S rDNA transcription.

To reveal the consequence of the reduced 18S rRNA in ribosome biogenesis by loss of RCL1, we next performed polysome profile analysis using 15 DAP seeds from *rcl1* and WT plants. The result showed that the number of polysomes and 80S monosomes decreased in *rcl1*, and the peak corresponding to the free 40S small subunit pool was nearly undetectable, whereas the free 60S large subunit peak increased, compared with that in the WT plants (Figure 7G). These results together reflected a deficit in mature 18S rRNA production and 40S ribosomal subunits, as well as a ribosomal subunit imbalance in *rcl1* seeds.

### Loss of RCL1 function impairs 18S pre-rRNA processing

**Figure 8:**
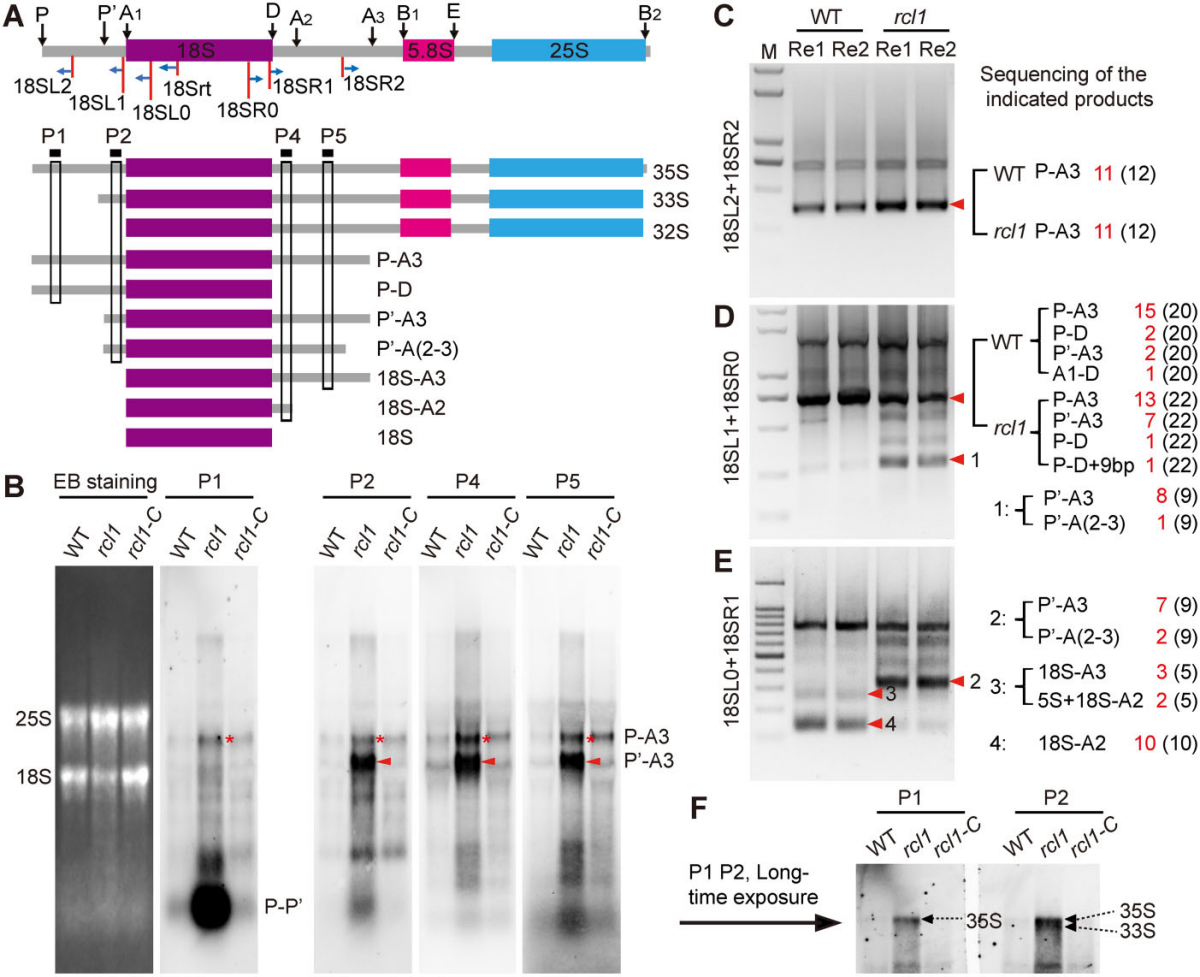
Loss of *RCL1* function affects 18S pre-rRNA processing. A, Diagram illustrating the 35S pre-rRNA structure, various cleavage sites, the pre-rRNA processing intermediates, the locations of RNA gel blots probes and the primers used for circular RT-PCR analysis. Black arrows above the diagram indicate cleavage sites. B, RNA gel blot analysis of 18S pre-rRNAs accumulating in the WT, *rcl1* and complemented *rcl1* line (*rcl1-C*) at 15 DAP. Equal amounts of total RNA were loaded and transferred to different membranes subjected to hybridization with probes as indicated in (A). Ethidium bromide staining (EB stain) of a membrane is shown (leftmost) as a loading control. C, Circular RT-PCR analysis of P-A3 intermediate. Primer location was indicated in (A). The cDNA was reverse transcribed with the 18Srt specific primer. D and E, Identification of the 5′ (D) and 3′ (E) terminals of 18S pre-rRNAs by circular RT-PCR. Primer’s location was indicated in (A). Red triangle pointed to pre-rRNA intermediates analyzed by clone sequencing. The results were listed on the right spaces. F, Long time exposure picture of P1 and P2 hybridization as in (B) showing increased 35S and 35S/33S intermediates in *rcl1*. In (C–E), numbers in the brackets mean total clones sequenced and the fonts in red indicate the corresponding fragments before. Re1 and Re2 represent two replicated RNA samples from different ears.

The maturation of 18S rRNA requires proper removal of the 5′-ETS and ITS1 sequence located at 5' and 3' by exoribonuclease and endoribonucleases at specific sites (Liu et al., 2020). In the 35S pre-rRNA, the 5′-ETS contains P' and A1 sites and the ITS1 contains D, A_2_, A_3_, and B_1_ sites (Supplemental Figure S1). The observation that several bands (Figure 7E) amplified by 18S mature rRNA-specific primers accumulated in *rcl1* mutants implied that the cleavage of the 5′-ETS and/or ITS1 was impaired in these mutants. To explore the processing steps of 18S rRNA maturation that are affected by *RCL1* mutation, different oligonucleotide probes (P1–P5) were synthesized and RNA gel blot analyses were performed to detect the steady-state levels of 18S pre-rRNA intermediates (Figure 8A). Hybridization with P1, which is complementary to a region upstream of the P'site, revealed that accumulated pre-rRNA extends from the P site to A_3_ (P-A3). This RNA was also detected with probes P2, P4, and P5 (Figure 8B), which are complementary to regions from site A_1_ to A_3_. In addition, the striking accumulation of the P '-A3 product, visualized with probes P2, P4, and P5, but not P1, probably represented the intermediate extending from P' to A_3_ site. The P-A3 product was generated by cleavage of 35S pre-rRNA at the A_3_ site in the ITS1 first pathway; further cleavage of P-A3 at the P' site generates P‘-A3. The overaccumulation of P-A3 and P’-A3 suggest that RCL1 participates in 35S pre-rRNA processing at the P′, A_1_, and A_2_ sites. We detected much more P′-A3 pre-rRNA than P-A3 with the P2, P4, and P5 probes (Figure 8B), indicating that processing at the P' site is less affected than at sites A_1_ and A_2_ in *rcl1*.

We carried out several PCR amplifications (Supplemental Figure S8B) with circularized cDNA to confirm the sites that failed to get processed during 18S rRNA maturation and the nature of the accumulated bands detected by RNA gel blots in *rcl1*. Consistent with our RNA gel blot results, we found increased P-A3 pre-rRNA in *rcl1* kernels compared with the WT using the 18SL2 and 18SR2 primers (Figure 8C). We next applied the 18SL1 and 18SR0 primers to compare the processing of 5'-ETS. Electrophoresis of the amplification products revealed an increased band (designated as product 1) in *rcl1* (Figure 8D). Sequencing of this product showed that it was mainly composed of P'-A3 intermediates (8 of 9) and P'-A (2–3) (1 of 9, Figure 8D). To validate the 3'-end cleavage deficiency, we amplified the intermediates with the primer pair 18SL0 + 18SR1, which produced an intermediate product (Product 2) analogous to Product 1, and we validated Product 2 by sequencing (Figure 8E). Additionally, two bands (Products 3 and 4) showed reduced accumulation in *rcl1*. Sequence analysis revealed that product 4 was composed of a 18S-A2 intermediate (10/10) and that Product 3 was composed of 18S-A3 and unexpected heteromorphy of 5S-18S-A2 (Figure 8E).

In maize, cleavage at the P', A_1_ and A_2_ sites is also required for processing in the 5'-ETS pathway. Cleavage of 33S at A_1_ site yields 32S pre-rRNA, and subsequent processing at A_2_ splits the small subunit rRNAs (18S-A2) and large subunit rRNAs intermediate (A2-B2, Supplemental Figure S1). After a long exposure, RNA gel blot analyses using P1 and P2 probes revealed increased 35S and 35S/33S intermediates in *rcl1* kernels (Figure 8F). We next designed primer pairs that allow the detection of 35S, 33S, and 32S pre-rRNAs by circular RT-PCR analysis (Supplemental Figure S9A). After amplification, we found increased 35S and 33S pre-rRNAs, but reduced 32S and in *rcl1* kernels, compared with WT (Supplemental Figure S9B), suggesting impaired cleavage at the A_1_ site. No evident product corresponding to A2-B2 could be detected under our experimental conditions (Supplemental Figure S9, C and D). We did not observe decreased pre-rRNA intermediates (such as A3-B2 and B1-B2) for 5.8S and 25S rRNA, indicating a neglectable role of RCL1 in the processing of 5.8S and 25S rRNA (Supplemental Figures S1 and S9D). These data demonstrated essential roles for RCL in both 5'-ETS and ITS1 cleavage during 18S pre-rRNA processing. We conclude that RCL1 participates in processing at the P', A_1_ and A_2_ sites for both the ITS1-first and 5 '-ETS-first pathways.

### Protein translation is inhibited in *rcl1* seeds

**Figure 9:**
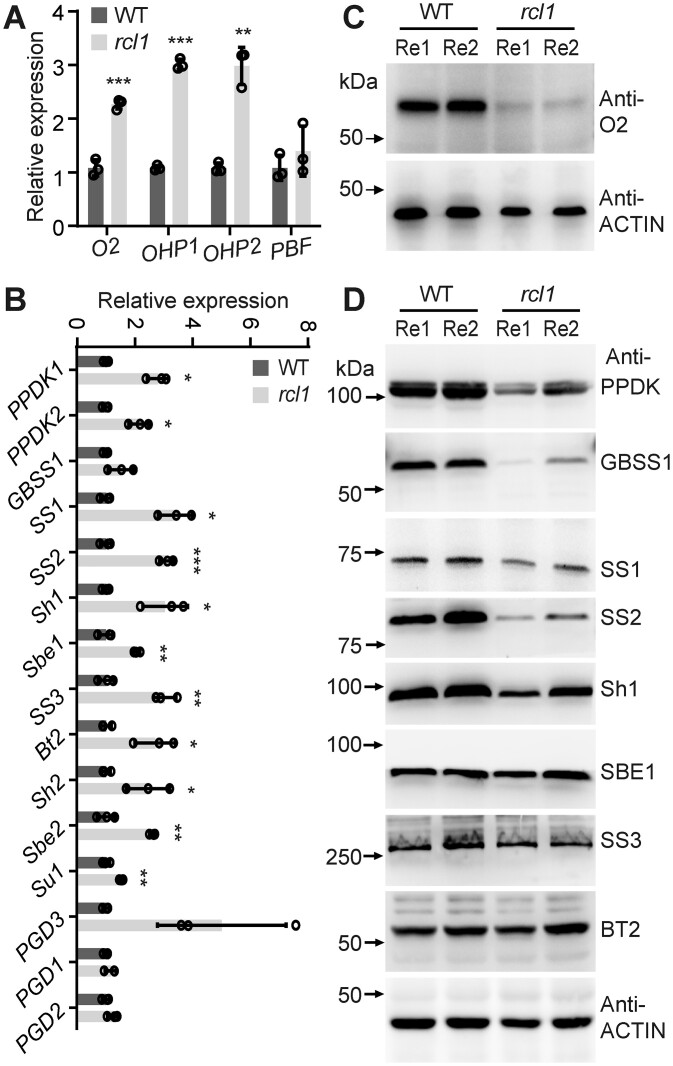
Analysis of transcript and protein of zein gene regulators and starch-related enzymes. A and B, Quantitative RT-PCR showing relative expressions of zein gene regulators (A) and starch-related enzymes (B) in the WT and *rcl1*. The data shown were obtained from three biological replicates from different ears. Error bars represent ±sd. ^*^P < 0.05, ^**^P < 0.01, and ^***^P < 0.001, Student’s *t* test. C and D, Immunoblots for protein accumulations of O2 (C) and starch-related enzymes (D) in the WT and *rcl1* seeds. Non-zein proteins from 15-DAP kernels of WT and *rcl1* were subjected to immunoblot analysis. The ACTIN antibody was used as a loading control. Re1 and Re2 represent two replicated proteins from different ears.

Starch and proteins are the primary storage reserves contributing to kernel weight. In contrast to the complex non-zein proteins, maize zein proteins and their regulators are well studied (Li and Song, 2020). To determine the mechanism of reduced zein protein contents in *rcl1*, we first analyzed the expression of *zein* genes and their known regulators. The RT-qPCR showed that the expression of *zein* genes in *rcl1* was generally downregulated (Supplemental Figure S9A), consistent with reduced zein accumulation. At the same time, the transcription factor genes *Opaque2* (*O2*), *Opaque2 heterodimerizing protein1* (*OHP1*), and *OHP2* were significantly upregulated, compared with that in the WT (Figure 9A). We next analyzed the O2 protein level because of its predominant role in zein transcription and the availability of its antibodies. Immunoblotting using non-zein from 15-DAP endosperm showed that O2 protein was present at an undetectable level in *rcl1* (Figure 9C). The reduced level of O2 is consistent with the down-regulated expression of 19 and 22 α-zein.

The enzymes for starch synthesis have also been well characterized (Keeling and Myers, 2010). We next performed RT-qPCR to analyze the expression of reported starch synthesis genes. In *rcl1*, *pyruvate orthophosphate dikinase1* (*PPDK1*), *PPDK2*, *sucrose synthase1* (*SS1*), *SS2*, *SS3*, *Shrunken1* (*Sh1*), *Sh2*, *Sugary1* (*Su1*), *Starch branching enzyme1* (*Sbe1*), *Sbe2*, *6-phosphogluconate dehydrogenase3* (*PGD3*), and *brittle endosperm2* (*Bt2*) transcript levels were significantly increased, while no significant increase of *Granule-bound starch synthase1* (*GBSS1*), *6-phosphogluconate dehydrogenase1* (*PGD1*), *PGD2*, and *PGD3* transcripts were detected in comparison to WT (Figure 9B). Although *PPDK1*, *PPDK2*, and *SS3* were shown to be transcriptionally activated by O2 (Zhang et al., 2016), the expression of these three genes was not affected by the striking decrease of O2, suggesting a possible complementary mechanism activated by other factors existed in *rcl1*. Contrary to the transcript levels, immunoblots showed the protein levels of PPDK, GBSS1, SS1, SS2, and Sh1 were lower than in WT, but no apparent change of SS3, SBE1, and Bt2 protein content was observed (Figure 9D). The discrepancy between transcript and protein levels suggested that mutation of *RCL1* may disrupt translation because of the reduced 18S rRNA and ribosome biogenesis.

To test whether protein translation in *rcl1* affected the amino acid contents, we next measured free amino acid levels by HPLC. Under our experimental conditions, sixteen amino acids were detected. No significant change in Arg and Lys contents was seen, but the remaining fourteen were significantly higher in *rcl1* compared with that in the WT (Supplemental Figure S10B). For example, the contents of Gly, Met, Phe, and His were about two-fold higher than the WT (Supplemental Table S2). These data indicated a decreased efficiency of amino acid utilization in *rcl1*, which agrees with the impaired protein translation process.

## Discussion

### Characterization of RNA 3′-terminal cyclase-like protein in plants

After transcription, newly synthesized cellular RNA molecules undergo modifications and maturation steps, during which enzymatic cleavage plays a crucial role. When cleaved, 3' termini of RNA generally contain a hydroxyl group, a phosphate, or a 2',3'-cyclic phosphate, either as processing intermediates or final products (Shigematsu et al., 2018). In addition to direct cleavage reactions by nucleases, an enzyme that directly acts at 3‘ RNA end producing cyclic phosphate was also discovered. Nearly three decades ago, RNA 3’-terminal phosphate cyclase (RTC) in human was first shown to convert the 3' phosphate into a 2',3'-cyclic phosphodiester in an ATP-dependent manner (Filipowicz et al., 1983; Reinberg et al., 1985). Subsequently, it became apparent that the RTC mediated cyclisation reaction occurs in three steps in this order: (1) formation of the covalent RTC-AMP intermediate, (2) transition of RNA-N^3^′p to RNA-N^3^′pp^5^′A and releasing RTC, and (3) generation of the cyclic phosphate through attacking the activated 3'-phosphate by the adjacent 2’-OH and releasing AMP (Genschik et al., 1997, 1998; Billy et al., 1999; Filipowicz, 2016; Shigematsu et al., 2018). Determination of the crystal structure of different RTC complexes and the covalent RTC AMP intermediate revealed the detailed processes and the essential amino acid residues required for the cyclisation reactions (Shimizu et al., 2008; Tanaka and Shuman, 2009; Tanaka et al., 2010; Desai et al., 2014). Despite these structural findings, the catalytical substrate RNA of RTCs in vivo and their biological functions are still ambiguous. Cloning of human RTC protein revealed that the human genome also contains a gene encoding RCL protein (Genschik et al., 1997), as do yeast and plant genomes (Figure 6B). It is noteworthy that plants have no genes coding for RTC family proteins in their genomes (Figure 6B). A possible explanation is that animal RTC proteins function in specialized tissues or distinct metabolic pathways that are not necessary or do not exist in plants.

Although maize RCL1 was predicted to contain a long cyclase domain occupying most of its protein sequence, it is unlikely that RCL1 functions as an RTC in catalyzing the formation of 2',3'-cyclic phosphate at the 3' RNA terminal. At least two reasons support this assumption. First, RCL1 diverges more extensively in its protein sequence with RTC from humans and *Escherichia coli*, compared with Rcl1p and human RCL1 (Figure 6B). Indeed, maize RCL1 lacks five of the seven conserved residues (Glu14, Arg43, Gln51, His52, and His320 in human) that are essential for the cyclisation reactions. Structural analysis showed that His309 (equivalent to His320 in human) of *E. coli* is responsible for the linkage between RTC and AMP to form the intermediate in the first step (Tanaka et al., 2010). Without His at this position, therefore, ZmRCL1 could not initiate the first step of the cyclization reaction. Second, RTC and ZmRCL1 exhibited entirely distinct subcellular localizations. Immunofluorescence of MYC-epitope tagged human RTC showed that nearly all signal (98%–99%) localized exclusively in the nucleoplasm and was excluded from the nucleoli (Genschik et al., 1997), whereas GFP fused RCL1 protein specifically targeted to nucleoli (Figure 6, C and D). Although there are no RTC orthologs in plants, it is still possible that plants also contain 2',3'-cyclic phosphate RNAs that are generated by many other kinds of enzymes, including endoribonucleases or ribozymes (Serganov and Patel, 2007; Hayashi et al., 2016; MacIntosh and Castandet, 2020).

### RCL1 function is conserved and involved in 18S pre-rRNA processing

In yeast, the primary 35S rRNA precursor (containing additionally 5′-ETS, 3′-ETS, ITS1, and ITS2 sequences) requires serial endonucleolytic cleavage to produce mature rRNA (Tomecki et al., 2017). Similar to maize (Supplemental Figure S1), cleavage of the 35S pre-rRNA at site A_2_ in the ITS1 produces 23S and 27S pre-rRNA for 18S, 5.8S, and 25S rRNA, respectively. Further cleavage at A_0_, A_1_, and A_2_ sites by endoribonucleases generates 20S pre-rRNA, which can be processed at the D site in the cytoplasm, yielding mature 18S rRNA (Kos and Tollervey, 2010; Tomecki et al., 2017). The yeast nucleolar protein Rcl1p, a putative nuclease, is required for the cleavage of 18S pre-rRNA. Depletion or inactivation of Rcl1p impairs pre-rRNA processing at A_0_, A_1_, and A_2_ sites, leading to a substantial decrease in the levels of 18S rRNA and 40S small ribosomal subunit (Billy et al., 2000; Horn et al., 2011).

Herein, we provide substantial evidence showing that maize RCL1 plays essential roles in the maturation of 18S rRNA, analogously to the yeast homolog Rcl1p. Like many ribosomal biogenesis factors (Palm et al., 2016; Montacié et al., 2017; Tomecki et al., 2017, Wells et al., 2017; Micol-Ponce et al., 2020), RCL1 is specifically localized to the nucleolus (Figure 6, C and D), where pre-rRNA processing takes place. More importantly, we observed altered levels of several 18S pre-rRNAs in *rcl1* mutants. Overaccumulation or reduction of a pre-rRNA intermediate suggests that it is the potential substrate or the product of the processing steps involved in ZmRCL1. Compared with WT, significantly increased levels of P-A3 and P′-A3 intermediates (Figure 8, B–E), the potential substrate for RCL1, were detected in *rcl1*. Consistently, we observed reduced amounts of the 18S-A3 or 18S-A2 intermediates in *rcl1* (Figure 8E), the subsequent products of the P′-A3 processed at A_1_ and A_2_ site, respectively (Supplemental Figure S1). Further evidence came from the altered levels of pre-rRNA in 5′-ETS first pathway, which requires cleavage at P′ and A_1_ at early processing stages (Supplemental Figure S1). In *rcl1*, 35S and 33S pre-rRNAs accumulated, while 32S pre-rRNA levels were reduced (Figure 8F and Supplemental Figure S9). These observations clearly revealed that RCL1 takes part in the cleavage at P′ (A_0_ in yeast) in 5′-ETS and at A_1_ and A_2_ sites in the ITS1, acting analogously to its yeast homolog Rcl1p. Accumulation of the 33S and P′-A3 pre-rRNA also indicates that mutation in RCL1 affected P′ cleavage to a lesser extent than at sites A_1_ and A_2_. Similar uncoupling of processing of P′ (A_0_ in yeast) from those at A_1_ and A_2_ sites have been demonstrated for mutations in Rcl1p (Billy et al., 2000). A possibility is that there is an additional conserved factor or processing mechanism that acts complementarily at the P′ site. Indeed, nucleolar protein RRP7 in Arabidopsis, the ortholog of yeast Rrp7, was demonstrated to participate in P′ site cleavage (Micol-Ponce et al., 2018). Thus, RRP7 in maize may promote P′ site cleavage in the absence of RCL1.

Ribosome biogenesis factors or ribonucleases for pre-rRNA processing are usually functionally conserved in eukaryotes. Mouse mRcl1 partially complemented the growth of the conditional *rcl1* strains on YPD (Billy et al., 2000). We demonstrated that ZmRCL1 and Rcl1p acted at similar processing sites for 18S rRNA maturation. However, ZmRCL1 failed to complement a conditional mutation of Rcl1p in yeast (Supplemental Figure S6B). This can be explained by the differing ITS1 sequence between yeast and maize. The cleavage site of A_2_ is slightly different in yeast (C
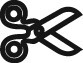
A) (Horn et al., 2011) and maize (A
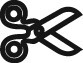
C) (Liu et al., 2020). Notably, the A_2_ site in yeast is located 213 bp downstream of the D site and maize A_2_ contains only 18 bp space between D and A_2_. The significantly more extended sequence from D to A_2_ in yeast pre-rRNA may produce a specific structure that cannot be recognized by ZmRCL1. In addition, the downstream flanking sequence of A_2_ is different between maize (UCUCCGC) and yeast (CACUGUG), and this sequence is essential for A_2_ cleavage (Horn et al., 2011). Therefore, identifying the structure or enzymatic activity of ZmRCL1 in the future will be a crucial step in elucidating its biochemical function in maize.

Even in the absence of RCL1, maize kernels can still produce a small amount of mature 18S rRNA (Figure 7) and produce storage zeins and functional proteins for the development of the aborted seeds (Figures 1 and 3). Reduced amounts of the 18S-A2 fragment detected by circular RT-PCR in *rcl1* indicated a decreased cleavage efficiency at the A_1_ and A_2_ sites (Figure 8E). Therefore, we considered that other processing factors could compensate for the processing at P′, A_1_ and A_2_ sites following the loss of RCL1. In addition to AtRRP7 for the P′ site, the PIN domain nuclease Utp24 in human and yeast was convincingly shown to participate in18S rRNA maturation through cleavage at sites A_1_ and A_2_ (Bleichert et al., 2006; Wells et al., 2016; An et al., 2018). Genomic analyses have identified a maize homolog of Utp24 (Jiao et al., 2017). Further investigation of the Utp24 for cleavage at the A_1_ and A_2_ sites is needed. Isolation of the Utp24 mutation and construction of *utp24 rcl1* double mutant in maize or isolation of a yet-unidentified nuclease will shed more light on 18S pre-rRNA processing in crop plants.

### Functions of RCL1 in developing kernels

Defective ribosome assembly and composition have emerged as significant causes of several serious diseases named ribosomopathies due to the highly variable clinical manifestations (Narla and Ebert, 2010; Armistead and Triggs-Raine, 2014; Kampen et al., 2020). Mutations in Arabidopsis genes coding for factors involved in 40S ribosome biogenesis showed defective seed phenotypes such as seed number reduction, reduced size (Missbach et al., 2013; Weis et al., 2015; Palm et al., 2019). In maize, mutations of genes involved in ribosome functions lead to abnormal kernel development and vegetative growth. *Urb2* mutation caused deficiencies in the 40S and 60S subunit and 80S ribosomes and increased ratios of polyribosomes, resulting in reduced seed size, lower plant height, and delayed tassel development (Wang et al., 2018). *Reas1* encodes an AAA-type ATPase protein controlling 60S large subunit export from the nucleus to the cytoplasm. Mutants of *Reas1* produce smaller kernels and show delayed development (Qi et al., 2016). In this study, we demonstrated that mutation of RCL1 induced serious embryo defects and a lethal phenotype. Compared with *urb2* and *reas1*, the lethality of *rcl1* suggested profound effects on ribosome formation caused by RCL1 mutation.

Depletion or inactivation of Rcl1p leads to a substantial decrease in 18S rRNA and 40S small ribosomal subunit levels, indicating an essential role for Rcl1p yeast cell viability (Billy et al., 2000; Horn et al., 2011). Rcl1 was also listed as one of the 315 essential genes for zebrafish development (Amsterdam et al., 2004). In maize, *rcl1* embryos failed to differentiate into leaf primordium, scutellum, and root primary primordium (Figure 2, A–E) and became degraded, indicating roles of RCL1-mediated 18S rRNA processing in embryogenesis and cell differentiation (Figure 1 and  Supplemental Figure S2A), although the detailed developmental defects of embryogenesis at the early stage in *rcl1* were not characterized. It was recently found that a stop-gain heterozygous mutation or copy number variants of *RCL1* gene in human (*hRCL1*) are associated with a range of neuropsychiatric phenotypes in human (e.g. catatonia, auditory and visual hallucinations, paranoia, aggression, mood dysregulation, and disorganized thoughts). In agreement with the neuropsychiatric phenotypes, hRCL1 is broadly expressed across several neuronal types, including enrichment within specific excitatory and inhibitory neuron clades, revealed by single-cell RNA sequencing from adult human neocortex (Brownstein et al., 2021). However, the normal growth and segregation of *rcl1/+* indicate that *RCL1* has no dose-specific effect and does not affect gametophytic development.

The essential roles of 18S rRNA processing and subsequent 40S ribosomal small subunit biogenesis for embryo development have been reported in Arabidopsis. Mutation in AtNOB1, an ortholog of a characterized endonuclease Nob1 in yeast, resulted in arrested embryo development at the globular stage. Recently, it was found that the nucleolar protein SAHY1 is involved in 18S pre-rRNA processing and embryos development in *sahy1* mutants arrested at the globular and heart stages, and torpedo stage. Because Arabidopsis only produces transient endosperm cells, it is uncertain if 18S rRNA processing is involved in later stages of endosperm development. Our observation of discontinuous BETL and abnormal aleurone cell identity suggested that RCL1 participates in both endosperm and embryo development.

Starches and proteins produced in the endosperm make the major contribution to seed weight and grain yield. The significant decrease in grain size is consistent with the decreased starch and their synthesized enzymes. Among the enzyme identified for starch synthesis in maize, at least five enzymes (PPDK, SS1 and SS2, GBSSI/Waxy, and Sh1) showed decreased protein levels in *rcl1*. Enzymes and proteins for starch synthesis usually associate with each other to form high molecular weight complexes (Hennen-Bierwagen et al., 2008). In *rcl1*, reduction of PPDK, SS1 and SS2, GBSSI/Waxy, and Sh1 may, therefore, disrupt the enzymatic complex formation and starch synthesis. Since that RCL1 mutation impairs 18S rRNA processing, essential for 40S small unit and protein translation, the discrepancy between the generally increased transcript and the reduced protein level could be attributed to reduced translation efficiency. We also found that zein protein synthesis was seriously delayed, and the content was remarkably decreased (Figure 3 and Supplemental Figure S3). Endosperm-specific bZIP transcription factor O2 binds to α-, β-, and γ- zein gene promoters to activate their expression (Li et al., 2015; Qiao et al., 2016; Yang et al., 2016). In *rcl1*, O2 protein becomes undetectable (Figure 9D). The accordance of reduced zein transcription and protein accumulation indicates both translational and transcriptional regulation. Therefore, it can be concluded that a slightly different mechanism leads to the reduced zein and starch phenotype in *rcl1*.

**Figure 10:**
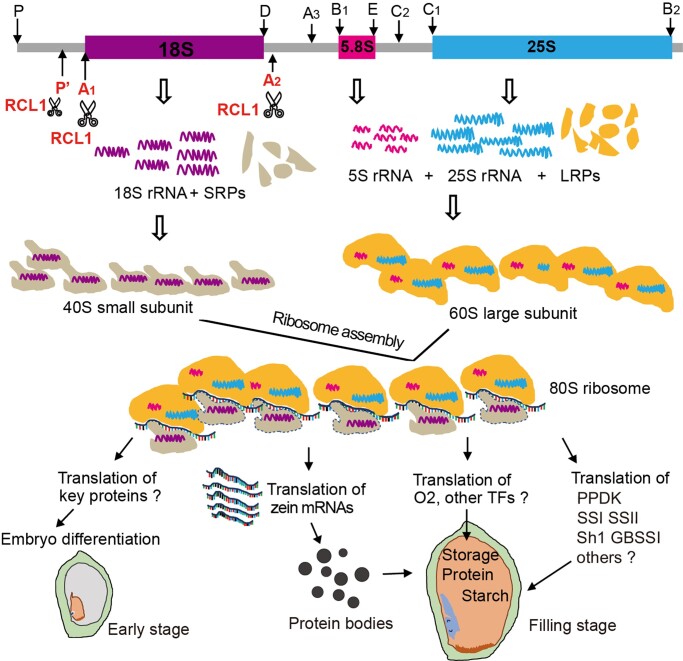
A proposed model for the role of RCL1. RCL1 participates in P′, A_1_ and A_2_ sites cleavage located in the 5′ and 3′ ends of mature 18S rRNA, which is essential for 40S small subunit maturation and mature 80S ribosome assembly. The resulting ribosomes translate proteins for embryo cell differentiation, zein proteins translation, and transcription factors and enzymes for storage reserve synthesis.

Apart from the well-defined starch synthesis enzymes and zein regulators, it is hard to attribute the apparent defects of embryo cell differentiation, BETL morphology and aleurone identities in *rcl1* to the translation of some specific genes given the complexity of pathways involved in cell differentiation. We proposed a working model for RCL1 during maize seed development (Figure 10). RCL1 promotes the pre-rRNA processing at the P′, A_1_, and A_2_ sites to generate 18S rRNA for ribosome small unit assembly. Mutation of RCL1 leads to decreased 18S rRNA, which impaired the 80S mature ribosome and polysome formation (Figure 7). During seed development, ribosomes translate critical functional proteins for embryogenesis and endosperm cell differentiation at the early stage. RCL1 mediated 18 rRNA maturation and ribosome assembly at the filling stage is essential for translating transcription factors such as O2, and multiple enzymes, involved in zein genes expression, translation and starch biosynthesis, respectively.

## Materials and methods

### Plant materials and growth

The Maize (*Z.* *mays*) *Mu1.7*-insertion mutant line UFMu-02204 and B73 inbred line were obtained from the UniformMu Stock Center (https://www.maizegdb.org/uniformmu) and Dr. Yongrui Wu, respectively. The CRISPR/Cas9 transgenic lines (in the C01 background) were created by Weimi company (Jiangsu province, China) through Agrobacterium (*Agrobacterium tumefaciens*)-mediated transformation methods. Maize plants were planted in the greenhouse of the experimental field in Hefei (Summer), in the field in Sanya (Winter) in China. *Nicotiana benthamiana* plants were grown in the greenhouse at Anhui Agricultural University at 23°C under a 16-h light and 8-h dark photoperiod using Philips LED tubs (90 μmol/m^2^/s).

### Analysis of *rcl1* seed phenotypes

For the germination assay, 20 WT and *rcl1* seeds were planted in the pots. The picture of seedlings was taken at 14 days after sowing. For kernel weight measurement, kernels from the central part of segregating ears were collected and numbered. The 100-kernel weight was quantified by an electronic balance. Kernel length, width and thickness were measured by a slide caliper. For longitudinal-section, seeds of WT and *rcl1* were harvested at maturity or 30 DAP and cut by single-edged cutting tool. The half seeds were directly observed via a stereomicroscope (Leica). To distinguish live cells, the obtained 30 DAP seeds were then stained by 1% (w/v) 2,3,5-triphenyltetrazolium chloride at room temperature for two hours and observed. All the pictures of segregating ears and seedlings were taken by a digital camera (Canon EOS M6).

### Microscopic observation

For the paraffin sections, fresh *rcl1* and WT kernels were fixed in formalin–acetic acid–alcohol fixative (50% [v/v] ethanol, 5% [v/v] acetic acid, and 3.7% [v/v] formaldehyde), after which they were evacuated twice for 10 min with a vacuum pump and soaked in fresh FAA overnight. The fixed materials were dehydrated in a gradient of ethanol (50%, 60%, 70%, 85%, 95%, and 100% [v/v] ethanol in water) and a gradient of xylene solution (25%, 50%, 75%, and 100% xylene in ethanol [v/v]). The samples were then soaked six times in paraffin at 65°C for 12 h, after which they were embedded in a paraffin block. Thin sections (10 mm) were obtained using a microtome (Leica), which were then dewaxed in xylene and stained with toluidine blue. The images were observed and collected using a Leica microscope.

Transmission electron microscopy (TEM) observation was performed as previously described (Zhu et al., 2020). In brief, fresh endosperm was cut into small pieces and fixed in 2.5% glutaraldehyde (v/v) in 0.1 M phosphate buffer (pH 7.2) on ice. After washing several times, the endosperm was dehydrated through a series of acetone/water mixtures. Then the endosperms were embedded into and polymerized in molds (65°C) for 48 h. Ultrathin sections (50–70 nm thickness) were prepared and observed by TEM microscopy (JEOL, Japan). The scanning electron microscopy observations of the mature kernel from WT and *rcl1* were performed as described previously (Li et al., 2017; Yang et al., 2018).

### Protein and starch content measurement

Six dry seeds of WT and *rcl1* were ground into fine flour using steel beads. The fine flours were filtered with 80-mesh nylon and dried at 42°C over-night for starch content analysis. Total starch was measured with the Megazyme Assay Kit (Cat# K-TSTA) according to the manufacturer’s instructions.

Zein proteins were extracted from 100 mg flour by adding 1 mL of zein extraction buffer containing 70% ethanol, 2% 2-mercaptoethanol (v/v), 3.75 mM sodium borate (pH 10), and 0.3% SDS. After incubation at room temperature overnight (2 h for fresh endosperm), the mixtures were centrifuged for 15 min at 15,871 *g*. Then 100 μL zein protein supernatant was transferred to a new tube and 10 μL of 10% SDS was added. The obtained zein protein was dried using a Concentrator plus (Eppendorf) and finally dissolved in 100 μL of distilled water. For the non-zein extraction, the zein extraction buffer was poured out and the wash was repeated three times to remove as much zein as possible. Then, 1 mL non-zein extraction buffer (12.5 mM sodium borate, 5% SDS, 2% 2-mercaptoethanol [v/v]) was added, the sample was vortexed, and incubated at room temperature for 2 h. After centrifugation at 13,000 rpm for 15 min, non-zein proteins in the supernatant were obtained. SDS-PAGE analysis of zein and non-zein was performed on a 15% polyacrylamide gel. The protein concentration was measured using the BCA protein assay kit (Pierce, Cat# 23225), as described by Zhang et al, (2015). The concentration of total protein was calculated by the sum of zein and non-zein. Total protein content was also measured by the Dumas method (Maria, 2020).

### Gene clone, allelic test, and genetic complementation

Heterozygous *rcl1*/+ plants were crossed to B73 to generate the F_1_ seeds. After self-pollination, 25 DAP endosperms of mutant seeds were collected for genomic DNA extraction and genotyping because of the clear phenotype. Map-based cloning was performed as described by Yang et al (2018). All the recombinants were identified using Indel markers listed in Supplemental Table S1. For CRISPR/Cas9 genome editing, the 20-bp gRNA targeted to the first exon selected and inserted in the transgenic vector. The genome-edited transgenic plants were validated by sequencing of the gRNA targeted area. Heterozygous plants were self-pollinated and crossed to *rcl1/+* ears to observe the kernel phenotype for the allelic test.

For genetic complementation, 3 × Flag coding sequence was added in the 5′ CDs of RCL1, and the resulted Flag-RCL1 fusion protein was driven by the *Ubi* promoter. After validation by PCR amplification, the transgenic pollen was crossed to *rcl1/+* ears to generate the F_1_ seed. We identified and self-pollinated F_1_ plants with *Mu1.7* insertion and Flag-RCL1 simultaneous. We next germinated the F_2_ progeny and identified complemented seeds with homozygous insertion for *Mu1.7* (*rcl1/-*) and *Flag-RCL1* transgene, as expected.

### Phylogenetic analysis and sequence alignment

Homologs of ZmRCL1 in various species were obtained using the NCBI protein BLAST tool. The phylogenetic tree was constructed using the neighbor-joining method within the MEGA version 6 software (www.megasoftware.net). Default parameters are Poisson correction, pairwise deletion, and bootstrap (1,000 replicates; random seed). Full multiple sequence alignments were generated using Bio-Edit software and ClustalW program (Thompson, et al., 1994). A graphic view with a 100% or 70% threshold was displayed as black and grey boxes, indicating identical or conserved amino acids, respectively.

### Subcellular localization and co-localization

To determine the subcellular localization of RCL1, we generated two constructs: p35S-RCL1-GFP (full-length CDs of RCL1) and p35S-RRP7-RFP (full-length CDs of AtRRP7). The fragments were amplified from cDNA prepared from 10 DAP B73 endosperm and *A.* *thaliana* leaves. The resulting PCR products were cloned into the *Bam*H I and *Sal* I (RCL1) sites of pCAMBIA1300-35S-GFP, or *Kpn* I and *Xba* I (AtRRP7) sites of pCAMBIA1300-35S-RFP to create fusion proteins. The plasmids were transferred into *Agrobacterium tumefaciens* (GV3101 strain) and injected into 3-week-old *N.* *benthamiana* leaves. Two days later, the injected areas were observed using an LSM880 confocal microscope (Zeiss, Jena, Germany). For ZmRCL1 subcellular localization in maize, the constructs of 35S-RCL1-GFP and 35S-RRP7-RFP were co-transformed into maize endosperm protoplasts using polyethylene glycol-mediated transformation as previously described (Yoo et al., 2007). The B73 endosperm protoplasts were isolated at eight DAP. The transformed protoplasts were cultured at 25°C in the dark for 36 h, and fluorescence was observed via confocal microscopy using an LSM880 confocal microscope (Zeiss, Jena, Germany).

### RNA in situ hybridization

In situ hybridization was performed according to protocols as previously described (Zhao et al., 2010). The maize kernels at different periods after pollination were harvested and fixed in FAA for 16 h. The seeds were dehydrated with a graded ethanol series (50%–100% [v/v]), embedded in paraffin (Sangon Biotech, Shanghai, China) and sliced into 10 mm sections by microtome (Leica). Templates of RNA probes were amplified from cDNAs with gene-specific primers containing the T7 promoter sequence at the 5′ or 3′ ends. RNA probes were synthesized using DIG RNA Labeling Kit (Roche, Basel, Switzerland). After blotting with Anti-digoxigenin AP-conjugate (Roche) and incubation with the NBT solution (Roche), the sections were observed and photographed with a Leica microscope.

### RNA extraction, RT-qPCR, circular RT-PCR, and RNA gel blot

Total RNA was extracted from 15 DAP seeds of WT and the *rcl1* mutant with Fast-Pure plant total RNA isolation kit (polysaccharides/polyphenolics-rich, Vazyme, Nanjing, Jiangsu, Chaina, cat# RC401-01). For quantitative real-time PCR, 2 μg of total RNA was reverse transcribed to complementary DNA (cDNA) using 1st Strand cDNA Synthesis Kit (+gDNA wiper) (Vazyme, cat# R301-01). All RT-qPCR analyses were performed using qPCR SYBR Green Mix (Vazyme, cat# Q111-02) on a Bio-Rad CFX-96 PCR thermocycler. The relative gene expression was normalized using *Ubi* (GRMZM2G409726) as a reference. Three biological replicates from different ears were performed for each analysis. Relative quantifiable differences in gene expression were analyzed as described previously (Schmittgen and Livak, 2008).

For Agilent RNA 2100 assay, the extracted RNA samples were diluted into 500 ng/μL and 1 μL total RNA was loaded. The assay was performed at LC Bio (Hangzhou, Zhejiang, China). Circular RT-PCR analysis was performed as described previously. The extracted 5 μg total RNAs were circularized by T4 RNA ligase 1 (NEB, Hamilton, MA, USA, cat# M0204) according to the provided protocol. The 18Srt and 5.8Srt primers (Supplemental Table S1) were used for reverse transcription of circularized precursors of 18S and 5.8S rRNA, respectively. After amplification for 35 cycles with 100 ng cDNA templet and the indicated primer pairs (Supplemental Figure S8B), the obtained products were analyzed by 1.5% (W/V) agarose gel. For sequencing, the bands were cloned into the pBlunt-Zero vector (TRANSGENE, Beijing, China, cat# CB501-01), and multiple clones were sequenced using M13F primer.

For RNA gel blot analysis, 7 μg of total RNA was separated on a 1.85%–2% (W/V) agarose/formaldehyde gel and transferred to a Hybond N+ membrane (GE Healthcare) by capillary elution. The probes were synthesized by GENERAL BIOL COMPANY (Chuzhou, Anhui, China). Electrophoresis, hybridization, and detection were performed as previously described (Jover-Gil et al., 2014). In brief, the membrane was prehybridized for 30 min at 45°C and hybridized overnight at 45°C with 4 μg of probes using a hybridization solution prepared by DIG Easy Hyb Granules (Roche). After washing in 2× SSC buffer (0.3 M NaCl, 30 mM sodium citrate) with 0.1% SDS once for 5 min at room temperature and twice for 10 min at 45°C, the membrane was transferred into blocking solution for 45 min. Blocking solution was prepared by Blocking Reagent (Roche, cat# 11096176001) with Maleic acid buffer consisting of 0.1 M Maleic acid-NaOH (pH 7.5), 0.15 M NaCl. Then the membrane was incubated for 45 min in with 0.05 U mL^−1^ α-Digoxigenin-Alkaline Phosphatase Fab fragments (Roche, cat# 11093274910), washed twice (15 min each) with washing buffer prepared by adding 3 mL Tween20 to maleic acid buffer, and equilibrated in detection buffer consisting of 0.1 M Tris–HCl (pH 9.0) and 0.1 M NaCl. At last, incubated with 25 mM CDP-Star (Roche, cat# 12041677001) for 5 min in the dark, the membrane was detected by Tanon-5200 imaging system (Tanon, Shanghai, China).

### Polysome profiling

For each sample, 20 15-DAP fresh kernels were peeled and kept in liquid nitrogen for 30 min. The kernels were then ground into fine powder with a mortar. An amount of 100 mg powder was hydrated in 300 μL lysis buffer containing 200 mM Tris–HCL (pH 9.0), 200 mM KCL, 25 mM EGTA, 35 mM Mg^2+^, 1% Brij-35, 1% Triton X-100, 1% Tween 20, 1% Igepal CA-630, 1% deoxycholic acid, 1% polyoxyethylene-10-tridecyl ether, 1 mM PMSF, 0.5 mg/mL heparin, 5 mM DTT, 50 μg/mL cycloheximide, 50 μg/mL chloramphenicol, and 40 U mL^−1^ RNase inhibitor. After incubation in the ice bath for 1 h, the mixtures were centrifuged for 10 min at 13,000 g. Then, 100 μL of supernatants were loaded onto a 10%–45% sucrose gradient. After ultracentrifugation at 36,000 rpm for 3 h at 4°C (Beckman Optima XE-100), the A_260_ absorbance was recorded using a piston gradient fractionator (Biocomp, Fredericton, Canada) equipped with a Bio-Rad Econo UV monitor.

### Immunoblotting

The non-zein protein was extracted from 15 DAP kernels of WT and the *rcl1* mutant kernels as described previously (Zheng et al., 2019). An amount of 15 μg of non-zein proteins were separated by 12% (w/v) SDS-PAGE gel and transferred to a Hydrophobic PVDF Transfer Membrane (Millipore, Burlington, MA, USA). Immunoblot analysis was performed as previously described. All of the primary antibodies of the starch synthesis pathway were related (Zhang et al., 2016). The mouse monoclonal anti-FLAG antibody (Sigma, St. Louis, MO, USA, Cat# F1804) and ACTIN antibody (Abclonal, Woburn, MA, Cat# AC009) were purchased. The secondary antibodies were goat anti-rabbit IgG-horseradish peroxidase (HRP) or goat anti-mouse IgG-HRP (Abmart, Berkeley Heights, NJ, USA, Cat# M21001L) and were diluted 1 : 5000 for performing the immune reaction. The signals were detected using the High-sig ECL Western Blotting Substrate (Tanon, Shanghai, China, Cat# 180-506) and were visualized using the Tanon-5200 imaging system (Tanon, Shanghai, China)

### HPLC analysis of free amino acid

About 100-mg fresh maize endosperm was triturated with steel balls and mixed with 1450 μL 4% (W/V) sulfosalicylic acid. After ultrasonic extraction for 30 min, the homogenate was allowed to stand for 10 min, and then the supernatant was taken out and centrifuged at the speed of 13,000 rpm for 40 min. The supernatant from the previous step was filtered through a 0.22 μm polyethersulfone membrane (Millipore, Burlington, MA, USA, cat# SLGP033R) and transferred to a 2 mL HPLC dedicated sample bottle. Finally, the amino acids were analyzed with a Hitachi-L8900 amino acid analyzer (Naka, Tokyo, Japan).

### Statistical analysis

All statistical tests analyses were conducted in GraphPad Prism version 8 (GraphPad Software, http://www.graphpad.com). A two-tailed Student’s *t* test was performed. The detailed statistical results are shown in Supplemental File S1.

## Accession numbers

*RCL1* (Zm00001d20853); *Ubi* (Zm00001d015327); O_2_ (Zm00001d018971); Pbf1 (Zm00001d005100); *OHP1* (Zm00001d034457); *OHP2* (Zm00001d013074); *PPDK1* (Zm00001d038163); *PPDK2* (Zm00001d010321); *GBSS1* (Zm00001d045462) *SS1(Zm00001d045261*), *SS2 (*Zm00001d002256); *SS3 (*Zm00001d026337); *Sh1 (Zm00001d045042*), *Sh2 (*Zm00001d044129); *Su1(*Zm00001d049753); *PGD1 (*Zm00001d035925), *PGD2(*Zm00001d042184); *PGD3(*Zm00001d049187); and *Bt2 (*Zm00001d050032). Protein sequence for phylogenetic can be found according to their NCBI Reference Sequence ID.

## Supplemental data

The following materials are available in the online version of this article.

**Supplemental Figure S1.** Diagram showing general processing procedure of rRNA in plants.

**Supplemental Figure S2.** Comparison of kernel phenotypes in WT and *rcl1* Mutants.

**Supplemental Figure S3.** Comparison of zeins and non-zein protein synthesis between the WT and *rcl1* developing kernels.

**Supplemental Figure S4.** Expression analysis of candidate genes and linkage analysis of *Mu1.7* insertion.

**Supplemental Figure S5.** Genetic complementation of *rcl1* mutant.

**Supplemental Figure S6.** Protein alignment of RCLs from plants.

**Supplemental Figure S7.** RCL1 is a homolog to yeast Rcl1p, but cannot complement its function.

**Supplemental Figure S8.** Sequencing and processing sites around the 18S rRNA.

**Supplemental Figure S9.** Primers and gel pattern of circular RT-PCR.

**Supplemental Figure S10.** Expression of zein encoding genes and measurement of free amino acid in WT and *rcl1* endosperm at 15 DAP.

**Supplemental Table S1.** Relative rRNA level in WT, *rcl1* and *rcl1-C* (% of total area).

**Supplemental Table S2.** Measurement of free amino acids content (μg/100 mg) in the endosperm from WT and *rcl1*.

**Supplemental Table S3.** Primers used in this study.

**Supplemental File S1.** Statistical analyses.

**Supplemental File S2.** Protein sequences for alignments.

## Supplementary Material

koac052_supplementary_data

## Notes

J.Y. conceived the project and designed the experiments. T.W. and J.Y. performed the majority of molecular experiments. Y.C. performed the mapping-based cloning. K.Z. completed the yeast transformation experiment. Q.D. helped with starch measurement and field planting. T.W. and J.Y. interpreted the data, generated the figures, and wrote the manuscript. J.Y. revised the manuscript.

Author responsibility

The author responsible for distribution of materials integral to the findings presented in this article in accordance with the policy described in the Instructions for Authors (https://academic.oup.com/plcell) is: Jun Yang (jyang@aheu.edu.cn).

## References

[koac052-B1] Ameismeier M , Zemp I, van den Heuvel J, Thoms M, Berninghausen O, Kutay U, Beckmann R (2020) Structural basis for the final steps of human 40S ribosome maturation. Nature 587**:** 683–687 33208940 10.1038/s41586-020-2929-x

[koac052-B2] Amsterdam A , Nissen RM, Sun Z, Swindell EC, Farrington S, Hopkins N (2004) Identification of 315 genes essential for early zebrafish development. Proc Natl Acad Sci USA 101**:** 12792–12797 15256591 10.1073/pnas.0403929101 PMC516474

[koac052-B3] An W , Du Y, Ye K (2018) Structural and functional analysis of Utp24, an endonuclease for processing 18S ribosomal RNA. PLoS ONE 13**:** e0195723 29641590 10.1371/journal.pone.0195723 PMC5895043

[koac052-B4] Armistead J , Triggs-Raine B (2014) Diverse diseases from a ubiquitous process: the ribosomopathy paradox. FEBS Lett 588**:** 1491–1500 24657617 10.1016/j.febslet.2014.03.024

[koac052-B5] Billy E , Hess D, Hofsteenge J, Filipowicz W (1999) Characterization of the adenylation site in the RNA 3'-terminal phosphate cyclase from *Escherichia coli*. J Biol Chem 274**:** 34955–34960 10574971 10.1074/jbc.274.49.34955

[koac052-B6] Billy E , Wegierski T, Nasr F, Filipowicz W (2000) Rcl1p, the yeast protein similar to the RNA 3'-phosphate cyclase, associates with U3 snoRNP and is required for 18S rRNA biogenesis. EMBO J 19**:** 2115–2126 10790377 10.1093/emboj/19.9.2115 PMC305690

[koac052-B7] Bleichert F , Granneman S, Osheim YN, Beyer AL, Baserga SJ (2006) The PINc domain protein Utp24, a putative nuclease, is required for the early cleavage steps in 18S rRNA maturation. Proc Natl Acad Sci USA 103**:** 9464–9469 16769905 10.1073/pnas.0603673103 PMC1480430

[koac052-B8] Boisvert FM , van Koningsbruggen S, Navascues J, Lamond AI (2007) The multifunctional nucleolus. Nat Rev Mol Cell Biol 8**:** 574–585 17519961 10.1038/nrm2184

[koac052-B9] Brownstein CA , Smith RS, Rodan LH, Gorman MP, Hojlo MA, Garvey EA, Li J, Cabral K, Bowen JJ, Rao AS, et al (2021) RCL1 copy number variants are associated with a range of neuropsychiatric phenotypes. Mol Psychiatry 26**:** 1706–1718 33597717 10.1038/s41380-021-01035-y PMC8159744

[koac052-B10] Carlson SJ , Shanker S, Chourey PS (2000) A point mutation at the Miniature1 seed locus reduces levels of the encoded protein, but not its mRNA, in maize. Mol Gen Genet 263**:** 367–373 10778757 10.1007/s004380051180

[koac052-B11] Cerezo E , Plisson-Chastang C, Henras AK, Lebaron S, Gleizes PE, O’Donohue MF, Romeo Y, Henry Y (2019) Maturation of pre-40S particles in yeast and humans. Wiley Interdiscip Rev RNA 10**:** e1516 30406965 10.1002/wrna.1516

[koac052-B12] Chen J , Zeng B, Zhang M, Xie S, Wang G, Hauck A, Lai J (2014) Dynamic transcriptome landscape of maize embryo and endosperm development. Plant Physiol 166**:** 252–264 25037214 10.1104/pp.114.240689 PMC4149711

[koac052-B13] Desai KK , Bingman CA, Cheng CL, Phillips GN Jr, Raines RT (2014) Structure of RNA 3'-phosphate cyclase bound to substrate RNA. RNA 20**:** 1560–1566 25161314 10.1261/rna.045823.114 PMC4174438

[koac052-B14] Doudna JA , Rath VL (2002) Structure and function of the eukaryotic ribosome: The next frontier. Cell 109**:** 153–156 12007402 10.1016/s0092-8674(02)00725-0

[koac052-B15] Fatica A , Oeffinger M, Dlakic M, Tollervey D (2003) Nob1p is required for cleavage of the 3 ' end of 18S rRNA. Mol Cell Biol 23**:** 1798–1807 12588997 10.1128/MCB.23.5.1798-1807.2003 PMC151717

[koac052-B16] Fernandez-Pevida A , Kressler D, de la Cruz J (2015) Processing of preribosomal RNA in *Saccharomyces cerevisiae*. Wiley Interdiscip Rev RNA 6**:** 191–209 25327757 10.1002/wrna.1267

[koac052-B17] Filipowicz W (2016) RNA 3′-terminal phosphate cyclases and cyclase-like proteins. Postepy Biochem 62**:** 327–334 28132487

[koac052-B18] Filipowicz W , Konarska M, Gross HJ, Shatkin AJ (1983) RNA 3′-terminal phosphate cyclase activity and RNA ligation in HeLa cell extract. Nucleic Acids Res 11**:** 1405–1418 6828385 10.1093/nar/11.5.1405 PMC325805

[koac052-B19] Fleming MB , Richards CM, Walters C (2017) Decline in RNA integrity of dry-stored soybean seeds correlates with loss of germination potential. J Exp Bot 68**:** 2219–2230 28407071 10.1093/jxb/erx100 PMC6055530

[koac052-B20] Fontanet P , Vicient CM (2008) Maize embryogenesis. Methods Mol Biol 427**:** 17–29 18369994 10.1007/978-1-59745-273-1_2

[koac052-B6426413] Foster J, , Kim HU, , Nakata PA, , Browse J (2012) A previously unknown oxalyl-CoA synthetase is important for oxalate catabolism in Arabidopsis. Plant Cell 24: 1217–1229 22447686 10.1105/tpc.112.096032 PMC3336115

[koac052-B21] Genschik P , Drabikowski K, Filipowicz W (1998) Characterization of the Escherichia coli RNA 3'-terminal phosphate cyclase and its sigma54-regulated operon. J Biol Chem 273**:** 25516–25526 9738023 10.1074/jbc.273.39.25516

[koac052-B22] Genschik P , Billy E, Swianiewicz M, Filipowicz W (1997) The human RNA 3'-terminal phosphate cyclase is a member of a new family of proteins conserved in Eucarya, Bacteria and Archaea. EMBO J 16**:** 2955–2967 9184239 10.1093/emboj/16.10.2955 PMC1169903

[koac052-B23] Gomez E , Royo J, Guo Y, Thompson R, Hueros G (2002) Establishment of cereal endosperm expression domains: identification and properties of a maize transfer cell-specific transcription factor, ZmMRP-1. Plant Cell 14**:** 599–610 11910007 10.1105/tpc.010365 PMC150582

[koac052-B24] Hang R , Wang Z, Deng X, Liu C, Yan B, Yang C, Song X, Mo B, Cao X (2018) Ribosomal RNA biogenesis and its response to chilling stress in *Oryza sativa*. Plant Physiol 177**:** 381–397 29555785 10.1104/pp.17.01714 PMC5933117

[koac052-B25] Hayashi S , Wakasa Y, Ozawa K, Takaiwa F (2016) Characterization of IRE1 ribonuclease-mediated mRNA decay in plants using transient expression analyses in rice protoplasts. New Phytol 210**:** 1259–1268 26831622 10.1111/nph.13845

[koac052-B26] Hennen-Bierwagen TA , Liu F, Marsh RS, Kim S, Gan Q, Tetlow IJ, Emes MJ, James MG, Myers AM (2008) Starch biosynthetic enzymes from developing maize endosperm associate in multisubunit complexes. Plant Physiol 146**:** 1892–1908 18281416 10.1104/pp.108.116285 PMC2287357

[koac052-B27] Henras AK , Plisson-Chastang C, O’Donohue MF, Chakraborty A, Gleizes PE (2015) An overview of pre-ribosomal RNA processing in eukaryotes. Wiley Interdiscip Rev RNA 6**:** 225–242 25346433 10.1002/wrna.1269 PMC4361047

[koac052-B28] Horn DM , Mason SL, Karbstein K (2011) Rcl1 protein, a novel nuclease for 18 S ribosomal RNA production. J Biol Chem 286**:** 34082–34087 21849504 10.1074/jbc.M111.268649 PMC3190816

[koac052-B29] Jiao Y , Peluso P, Shi J, Liang T, Stitzer MC, Wang B, Campbell MS, Stein JC, Wei X, Chin CS, et al (2017) Improved maize reference genome with single-molecule technologies. Nature 546**:** 524–527 28605751 10.1038/nature22971 PMC7052699

[koac052-B30] Jover-Gil S , Paz-Ares J, Micol JL, Ponce MR (2014) Multi-gene silencing in Arabidopsis: A collection of artificial microRNAs targeting groups of paralogs encoding transcription factors. Plant J 80**:** 149–160 25040904 10.1111/tpj.12609

[koac052-B31] Kampen KR , Sulima SO, Vereecke S, De Keersmaecker K (2020) Hallmarks of ribosomopathies. Nucleic Acids Res 48**:** 1013–1028 31350888 10.1093/nar/gkz637 PMC7026650

[koac052-B32] Keeling PL , Myers AM (2010) Biochemistry and genetics of starch synthesis. Annu Rev Food Sci Technol 1**:** 271–303 22129338 10.1146/annurev.food.102308.124214

[koac052-B33] Kos M , Tollervey D (2010) Yeast pre-rRNA processing and modification occur cotranscriptionally. Mol Cell 37**:** 809–820 20347423 10.1016/j.molcel.2010.02.024 PMC2860240

[koac052-B34] Lamanna AC , Karbstein K (2009) Nob1 binds the single-stranded cleavage site D at the 3'-end of 18S rRNA with its PIN domain. Proc Natl Acad Sci USA 106**:** 14259–14264 19706509 10.1073/pnas.0905403106 PMC2732849

[koac052-B35] Lange H , Zuber H, Sement FM, Chicher J, Kuhn L, Hammann P, Brunaud V, Bérard C, Bouteiller N, Balzergue S, et al (2014) The RNA helicases AtMTR4 and HEN2 target specific subsets of nuclear transcripts for degradation by the nuclear exosome in *Arabidopsis thaliana*. PLoS Genet 10: e1004564 25144737 10.1371/journal.pgen.1004564 PMC4140647

[koac052-B36] Leroux BM , Goodyke AJ, Schumacher KI, Abbott CP, Clore AM, Yadegari R, Larkins BA, Dannenhoffer JM (2014) Maize early endosperm growth and development: from fertilization through cell type differentiation. Am J Bot 101**:** 1259–1274 25104551 10.3732/ajb.1400083

[koac052-B69381781] Li G, , Wang D,, Yang R,, Logan K,, Chen H,, Zhang S,, Skaggs MI,, Lloyd A,, Burnett WJ,, Laurie JD,, Hunter BG, et al. (2014) Temporal patterns of gene expression in developing maize endosperm identified through transcriptome sequencing. Proc Natl Acad Sci USA 111: 7582–7587 24821765 10.1073/pnas.1406383111 PMC4040564

[koac052-B37] Li C , Song R (2020) The regulation of zein biosynthesis in maize endosperm. Theor Appl Genet 133**:** 1443–1453 31897513 10.1007/s00122-019-03520-z

[koac052-B38] Li C , Qiao Z, Qi W, Wang Q, Yuan Y, Yang X, Tang Y, Mei B, Lv Y, Zhao H, et al (2015) Genome-wide characterization of cis-acting DNA targets reveals the transcriptional regulatory framework of opaque2 in maize. Plant Cell 27**:** 532–545 25691733 10.1105/tpc.114.134858 PMC4558662

[koac052-B39] Li Q , Wang J, Ye J, Zheng X, Xiang X, Li C, Fu M, Wang Q, Zhang Z, Wu Y (2017) The maize imprinted gene floury3 encodes a PLATZ protein required for tRNA and 5S rRNA transcription through interaction with RNA polymerase III. Plant Cell 29**:** 2661–2675 28874509 10.1105/tpc.17.00576 PMC5774582

[koac052-B40] Liu GQ , Yan PS, Du QG, Wang YF, Guo Y, Fu ZY, Wang HQ, Tang JH (2020) Pre-rRNA processing and its response to temperature stress in maize. J Exp Bot 71**:** 1363–1374 31665749 10.1093/jxb/erz488

[koac052-B41] Lopes MA , Larkins BA (1993) Endosperm origin, development, and function. Plant Cell 5**:** 1383–1399 8281040 10.1105/tpc.5.10.1383 PMC160370

[koac052-B42] MacIntosh GC , Castandet B (2020) Organellar and secretory ribonucleases: Major players in plant RNA homeostasis. Plant Physiol 183**:** 1438–1452 32513833 10.1104/pp.20.00076 PMC7401137

[koac052-B43] Maria H (2020) Measuring protein content in food: An overview of methods. Foods 9: 1340 10.3390/foods9101340 PMC7597951 32977393

[koac052-B44] McCarty DR , Settles AM, Suzuki M, Tan BC, Latshaw S, Porch T, Robin K, Baier J, Avigne W, Lai J, et al (2005) Steady-state transposon mutagenesis in inbred maize. Plant J 44**:** 52–61 16167895 10.1111/j.1365-313X.2005.02509.x

[koac052-B45] Micol-Ponce R , Sarmiento-Manus R, Fontcuberta-Cervera S, Cabezas-Fuster A, de Bures A, Saez-Vasquez J, Ponce MR (2020) SMALL ORGAN4 is a ribosome biogenesis factor involved in 5.8S ribosomal RNA maturation. Plant Physiol 184**:** 2022–2039 32913045 10.1104/pp.19.01540 PMC7723108

[koac052-B46] Micol-Ponce R , Sarmiento-Manus R, Ruiz-Bayon A, Montacie C, Saez-Vasquez J, Ponce MR (2018) Arabidopsis RIBOSOMAL RNA PROCESSING7 is required for 18S rRNA maturation. Plant Cell 30**:** 2855–2872 30361235 10.1105/tpc.18.00245 PMC6305980

[koac052-B47] Missbach S , Weis BL, Martin R, Simm S, Bohnsack MT, Schleiff E (2013) 40S ribosome biogenesis co-factors are essential for gametophyte and embryo development. PLoS ONE 8: e54084 23382868 10.1371/journal.pone.0054084 PMC3559688

[koac052-B48] Montacié C , Durut N, Opsomer A, Palm D, Comella P, Picart C, Carpentier MC, Pontvianne F, Carapito C, Schleiff E, et al (2017) Nucleolar proteome analysis and proteasomal activity assays reveal a link between nucleolus and 26S proteasome in A. thaliana. Front Plant Sci 8**:** 1815 29104584 10.3389/fpls.2017.01815 PMC5655116

[koac052-B49] Narla A , Ebert BL (2010) Ribosomopathies: Human disorders of ribosome dysfunction. Blood 115**:** 3196–3205 20194897 10.1182/blood-2009-10-178129 PMC2858486

[koac052-B50] Nierhaus KH (2009) Nobel Prize for the elucidation of ribosome structure and insight into the translation mechanism. Angew Chem Int Ed Engl 48**:** 9225–9228 19899182 10.1002/anie.200905795

[koac052-B51] Palm D , Simm S, Darm K, Weis BL, Ruprecht M, Schleiff E, Scharf C (2016) Proteome distribution between nucleoplasm and nucleolus and its relation to ribosome biogenesis in *Arabidopsis thaliana*. RNA Biol 13**:** 441–454 26980300 10.1080/15476286.2016.1154252 PMC5038169

[koac052-B52] Palm D , Streit D, Shanmugam T, Weis BL, Ruprecht M, Simm S, Schleiff E (2019) Plant-specific ribosome biogenesis factors in *Arabidopsis thaliana* with essential function in rRNA processing. Nucleic Acids Res 47**:** 1880–1895 30576513 10.1093/nar/gky1261 PMC6393314

[koac052-B54] Qi W , Zhu J, Wu Q, Wang Q, Li X, Yao D, Jin Y, Wang G, Wang G, Song R (2016) Maize reas1 mutant stimulates ribosome use efficiency and triggers distinct transcriptional and translational responses. Plant Physiol 170**:** 971–988 26645456 10.1104/pp.15.01722 PMC4734584

[koac052-B55] Qiao Z , Qi W, Wang Q, Feng Y, Yang Q, Zhang N, Wang S, Tang Y, Song R (2016) ZmMADS47 regulates zein gene transcription through interaction with opaque2. PLoS Genet 12**:** e1005991 27077660 10.1371/journal.pgen.1005991 PMC4831773

[koac052-B56] Reinberg D , Arenas J, Hurwitz J (1985) The enzymatic conversion of 3'-phosphate terminated RNA chains to 2',3'-cyclic phosphate derivatives. J Biol Chem 260**:** 6088–6097 2581947

[koac052-B57] Sabelli PA , Larkins BA (2009) The development of endosperm in grasses. Plant Physiol 149**:** 14–26 19126691 10.1104/pp.108.129437 PMC2613697

[koac052-B58] Saez-Vasquez J , Delseny M (2019) Ribosome biogenesis in plants: From functional 45S ribosomal DNA organization to ribosome assembly factors. Plant Cell 31**:** 1945–1967 31239391 10.1105/tpc.18.00874 PMC6751116

[koac052-B59] Schmittgen TD , Livak KJ (2008) Analyzing real-time PCR data by the comparative CT method. Nat Protocol 3**:** 1101–1108 10.1038/nprot.2008.73 18546601

[koac052-B60] Serganov A , Patel DJ (2007) Ribozymes, riboswitches and beyond: Regulation of gene expression without proteins. Nat Rev Genet 8**:** 776–790 17846637 10.1038/nrg2172 PMC4689321

[koac052-B61] Shigematsu M , Kawamura T, Kirino Y (2018) Generation of 2',3'-cyclic phosphate-containing RNAs as a hidden layer of the transcriptome. Front Genet 9: 562 30538719 10.3389/fgene.2018.00562 PMC6277466

[koac052-B62] Shimizu S , Ohki M, Ohkubo N, Suzuki K, Tsunoda M, Sekiguchi T, Takenaka A (2008) Crystal structures of RNA 3'-terminal phosphate cyclase and its complexes with Mg^2+^ +ATP, ATP or Mn^2^^+^. Nucleic Acids Symp Ser (Oxf) 52: 221–222 10.1093/nass/nrn112 18776333

[koac052-B63] Tanaka N , Shuman S (2009) Structure-activity relationships in human RNA 3'-phosphate cyclase. RNA 15**:** 1865–1874 19690099 10.1261/rna.1771509 PMC2743044

[koac052-B64] Tanaka N , Smith P, Shuman S (2010) Structure of the RNA 3'-phosphate cyclase-adenylate intermediate illuminates nucleotide specificity and covalent nucleotidyl transfer. Structure 18**:** 449–457 20399182 10.1016/j.str.2010.01.016 PMC2858066

[koac052-B65] Tomecki R , Sikorski PJ, Zakrzewska-Placzek M (2017) Comparison of preribosomal RNA processing pathways in yeast, plant and human cells—Focus on coordinated action of endo- and exoribonucleases. FEBS Lett 591**:** 1801–1850 28524231 10.1002/1873-3468.12682

[koac052-B66] Thompson JD , Higgins DG, Gibson TJ (1994) CLUSTAL W: Improving the sensitivity of progressive multiple sequence alignment through sequence weighting, position-specific gap penalties and weight matrix choice. Nucleic Acids Res 22**:** 4673–4680 7984417 10.1093/nar/22.22.4673 PMC308517

[koac052-B67] Walley JW , Sartor RC, Shen Z, Schmitz RJ, Wu KJ, Urich MA, Nery JR, Smith LG, Schnable JC, Ecker JR, et al (2016) Integration of omic networks in a developmental atlas of maize. Science 353**:** 814–818 27540173 10.1126/science.aag1125 PMC5808982

[koac052-B68] Wang H , Wang K, Du Q, Wang Y, Fu Z, Guo Z, Kang D, Li WX, Tang J (2018) Maize Urb2 protein is required for kernel development and vegetative growth by affecting pre-ribosomal RNA processing. New Phytol 218**:** 1233–1246 29479724 10.1111/nph.15057

[koac052-B69] Weis BL , Kovacevic J, Missbach S, Schleiff E (2015) Plant-specific features of ribosome biogenesis. Trends Plant Sci 20**:** 729–740 26459664 10.1016/j.tplants.2015.07.003

[koac052-B70] Wells GR , Weichmann F, Colvin D, Sloan KE, Kudla G, Tollervey D, Watkins NJ, Schneider C (2016) The PIN domain endonuclease Utp24 cleaves pre-ribosomal RNA at two coupled sites in yeast and humans. Nucleic Acids Res 44: 9016 27418679 10.1093/nar/gkw645 PMC5062985

[koac052-B71] Wells GR , Weichmann F, Sloan KE, Colvin D, Watkins NJ, Schneider C (2017) The ribosome biogenesis factor yUtp23/hUTP23 coordinates key interactions in the yeast and human pre-40S particle and hUTP23 contains an essential PIN domain. Nucleic Acids Res 45**:** 4796–4809 28082392 10.1093/nar/gkw1344 PMC5416842

[koac052-B72] Wilson DN , Doudna Cate JH (2012) The structure and function of the eukaryotic ribosome. Cold Spring Harb Perspect Biol 4: a011536 22550233 10.1101/cshperspect.a011536 PMC3331703

[koac052-B73] Yang J , Ji C, Wu Y (2016) Divergent transactivation of maize storage protein zein genes by the transcription factors opaque2 and OHPs. Genetics 204**:** 581–591 27474726 10.1534/genetics.116.192385 PMC5068848

[koac052-B74] Yang J , Fu M, Ji C, Huang Y, Wu Y (2018) Maize oxalyl-CoA decarboxylase1 degrades oxalate and affects the seed metabolome and nutritional quality. Plant Cell 30**:** 2447–2462 30201823 10.1105/tpc.18.00266 PMC6241262

[koac052-B75] Yoo SD , Cho YH, Sheen J (2007) Arabidopsis mesophyll protoplasts: A versatile cell system for transient gene expression analysis. Nat Protoc 2**:** 1565–1572 17585298 10.1038/nprot.2007.199

[koac052-B76] Zhan J , Thakare D, Ma C, Lloyd A, Nixon NM, Arakaki AM, Burnett WJ, Logan KO, Wang D, Wang X, et al (2015) RNA sequencing of laser-capture microdissected compartments of the maize kernel identifies regulatory modules associated with endosperm cell differentiation. Plant Cell 27**:** 513–531 25783031 10.1105/tpc.114.135657 PMC4558669

[koac052-B77] Zhang SS , Zhan JP, Yadegari R (2018) Maize opaque mutants are no longer so opaque. Plant Reprod 31**:** 319–326 29978299 10.1007/s00497-018-0344-3 PMC6105308

[koac052-B78] Zhang XR , Qin Z, Zhang X, Hu Y (2015) Arabidopsis SMALL ORGAN 4, a homolog of yeast NOP53, regulates cell proliferation rate during organ growth. J Integr Plant Biol 57**:** 810–818 26310197 10.1111/jipb.12424

[koac052-B79] Zhang Z , Zheng X, Yang J, Messing J, Wu Y (2016) Maize endosperm-specific transcription factors O2 and PBF network the regulation of protein and starch synthesis. Proc Natl Acad Sci USA 113**:** 10842–10847 27621432 10.1073/pnas.1613721113 PMC5047157

[koac052-B80] Zhao Z , Andersen SU, Ljung K, Dolezal K, Miotk A, Schultheiss SJ, Lohmann JU (2010) Hormonal control of the shoot stem-cell niche. Nature 465**:** 1089–U1154 20577215 10.1038/nature09126

[koac052-B81] Zheng XX , Li Q, Li CS, An D, Xiao Q, Wang WQ, Wu YR (2019) Intra-kernel reallocation of proteins in maize depends on VP1-mediated scutellum development and nutrient assimilation. Plant Cell 31**:** 2613–2635 31530735 10.1105/tpc.19.00444 PMC6881121

[koac052-B82] Zhu J , Lou Y, Shi QS, Zhang S, Zhou WT, Yang J, Zhang C, Yao XZ, Xu T, Liu JL, et al (2020) Slowing development restores the fertility of thermo-sensitive male-sterile plant lines. Nat Plants 6**:** 360–367 32231254 10.1038/s41477-020-0622-6

