# Supplementary material for: Maize RNA 3'-terminal phosphate cyclase-like protein promotes 18S pre-rRNA cleavage and is important for kernel development
Source: Plant Cell. 2022 Feb 15;34(5):1957–79. doi: 10.1093/plcell/koac052 (PMC9048941; doi:10.1093/plcell/koac052)
Supplement: koac052_supplementary_data [file koac052_supplementary_data.zip › Supplemental File 2.pdf]

Protein sequences for alignments:

>ZmRCL1 (*Zea mays*) NP_001130728.1
MGRDKSRRLYGSRHFRQRLVLATLTSTAVTIEDIRSGDASPGLRPYEVSLLRLLDKISDHH
TIDLNETGTKLRYRPGVIIGGKGLEHDCGVHRGIGYFLEPLILLGLFSRAPISIRLKGITNDT
KDPSVDTFRTTTLHMLKHFGVPLDGFDLNIDSRGSPPLGGGEVFLRVRNINSTLTAANW
VDEGMVKRIRGVSFSTRVSPQLENRIIYAARGIFNRFIPDVHIHTDHRSGSAGGRSPGYG
VSLVAETTTGCLLSVDVTVSYPSVDEINEESEKPELTSPEDLGVQAASMLLEEVAQGGVV
DSTHQGLLFILCALCPPDVSKVRVGQLTPYGIETLRNIRDFLDVKFIIKPDPNSNTVTLKCV
GAGVKNLARKIS

>HsRCL1 (*Homo sapiens*) NP_005763.3
MATQAHSLSYAGCNFLRQRLVLSTLSGRPVKIRKIRARDDNPGLRDFEASFIRLLDKITNG
SRIEINQTGTTLYYQPGLLYGGSVEHDCSVLRGIGYYLESLLCLAPFMKHPLKIVLRGVTN
DQVDPSVDVLKATALPLLKQFGIDGESFELKIVRRGMPPGGGGEVVFSCPVRKVLKPIQL
TDPGKIKRIRGMAYSVRVSPQMANRIVDSARSILNKFIPDIYIYTDHMKGVNSGKSPGFGL
SLVAETTSGTFLSAELASNPQGQGAAVLPEDLGRNCARLLLEEIYRGGCVDSTNQSLALL
LMTLGQQDVSKVLLGPLSPYTIEFLRHLKSFFQIMFKIETKPCGEELKGGDKVLMTCVGI
GFSNLSKTLK

>ScRCL1p (*Saccharomyces cerevisiae*) AJT71928.1
MSSSAPKYTTFQGSQNFRLRIVLATLSGKPIKIEKIRSGDLNPGLKDYEVSFLRLIESVTN
GSVIEISYTGTTVIYRPGIIVGGASTHICPNSKPVGYFVEPMLYLAPFSKKKFSILFKGITAS
HNDAGIEAIKWGLMPVMEKFGVRECALHTLKRGSPPLGGGEVHLVVDSLIAQPITMHEID
RPIISSITGVAYSTRVSPSLVNRMIDGAKKVLKNLQCEVNITADVWRGENSGKSPGWGITL
VAQSKQKGWSYFAEDIGDAGSIPEELGEKVACQLLEEISKSAAVGRNQLPLAIVYMVIGK
EDIGRLRINKEQIDERFIILLRDIKKIFNTEVFLKPVDEADNEDMIVTIKGIGFTNTSKKIA

>SbRCL1 (*Sorghum bicolor*) XP_002460403.1
MGRDKSRRLSGSRDFRQRLVLATLTSTAVTIVDIRSGDAAPGLRPHEVSLLRLLDKISDH
HTIELNDTGTKLKYRPGVIIGGKGLEHDCGVHRGIGYFLEPLILLGLFARAPISIRLKGITND
TKDPSVDTFRTTTLHMLKHFGVPLEGFDLKIDSRGSPPLGGGEVSLWVRNINSTLTAAN
WVDEGMVKRIRGVSFATRVSPQLQSRIIYAARGIFNRFIPDVHIDKDHRSGSAGGRSPGY
GVTLVAETTTGCLLSVDVTVSCPSVDEINEESEKPELTSPEDLGVQAASMLLEEVAQGGV
VDSTHQGLLFILCALCPPDVSKVRVGQLTPYGIETLRNIRDFLDVKFIIKPDPNSNTVTLKC
VGAGVKNLARKIS

>OsRCL1 (*Oryza sativa* Japonica Group) XP_015628085.1
MGRDKARRLSGSRHFRQRVVLATLTSTAITIDDIRSGGAAPGLRPHEVSLLHLLHKISDHH
SLDLNETGTKLRYKPGVIVGGRDLEHDCGVHRGIGYFLEPLILLGLFARAPISIRLKGITND
TKDPSVDTFRMVTLHMLKQFGVPLEGLELKIESRGAPPLGGGEVFLRVPNIKSTLKAAN
WADEGMVKRIRGVSFSTRVSPQIENRIIYAARGIFNRFIPDVHIFTDHRSGSAGGRSAGY
GVSVVAETTTGCLLSADATVSYPNVDEMNEESENLELTSPEDLGVQVASMLLEEVAQGG
VVDSAHQGLLFILCALSPPDVSKVRVGQLTPYAIETLRNIRDFLDVKFIIKPDPNSNTVTLK
CVGAGVKNLARKTS

>BdRCL1 (*Brachypodium distachyon*) XP_003563054.1
MGRDKARRLTGSRHFRQRMVLATLTSTPIAIEDIRAGDAGLRAHEVSLLRLIDKISDHHSF
ELNETGTKLRYKPGVIVGGRDLEHDCGVHRGIGYFLEPLILLGLFSRSPISIRLKGITNDTK
DPSVDTFRMVTLHMLKHFGVPLEGLELKIENRGAPPLGGGEVHLRVPNINSTLTAANWID

EGMVKRIRGVTFSTRVSPQIENRILYAARGIFNKFIPDVHIFTDHRSGSSGGRSAGYGVS
VVAETTTGCLISADATVSYPNIDEMSKESEKPELMSPEDLGEQVAKMLLDEVAQGGVVD
STHQGLLFILCALCPPDVSRVRVGQLTPHAIETLRNIRDFLDVKFIIKPDPNSNTVTLKCVG
AGVKNLARKIS
>AtRCL1 (*Arabidopsis thaliana*) NP_680196.1
MVMMKKMKGSQSFRQRLLLSTLSSTPISIDEIRADETIPGLRPHEVNLLRLLEIVTDDAVV
DINETGTRLKYKPGTIVGGKNLVHSCSLSRSIGYYLEPLLLLGLFGKKPLSIRLKGITNDPR
DASVDTFRSTTLNIIKRFGVPAEDLELKIEARGVAPNGGGEVLLTVPNIKTLSAVHWVEEG
MVKKIRGTTFSTRVTSDFEHSMRFAARGIFNNLLPDVHIFQDHRAGAQAGKSPGYGISLA
AETTTGCFISADTTVSCERPDETGELDVEKKERSPAEDTGVEVASWLLQEIEKGGVVDS
THQGLLFLLCALSEQDVSKVRVGTLSPYAVETLRNIKEFLGVKFAIKPDPLTGTVILKCTGS
GLINLSRKLS
>GmRCL1 (Glycine max) NP_001242399.1
MGKTAYKRLKGSQSFRQRLLLSTLSSTPILIEDIRADETWPGLRNHEISLLRLFETVCDDC
HVEINETGTKLKYKPGTIMGGRQHSPHDCGVSRSIGYFLEPLIVLCLFAKQPLTIRLKGITN
DSKDPSVDTFKSTTLPILKRFGVPSEGLEIKVESRGLPPNGGGEVLLSVPVVQSLTAVSW
IDEGFVRKIRGTSFSTRVSVQFEYGMIKAARGIINPLVSDVHIFSDHRSGPEAGNSPGYGI
SLVAETTSGCFISVDTAVSQVRDDDTSGLADDAKRDLMSPDDIGVGIASALLGEIAQSGV
VDSTHQGLLFLLCALCPQDVSKVRVGKLSQHGVETLRNLRDFLDLKFIIKPDPNSQSVFL
KCIGYGMKNLSRKVS
>PpRCL1 (*Physcomitrium patens*) XP_024397674.1
MGGEVIKLRGAQQFRQRLLLATLAGATLRIDDIRAEDSRPGLRDYEASFLRLLEKVSNGC
SVEINETGTKIRYKPGVLIGGRDLVHDCGTGRSIGYFLESLLVLGLFGKKSLTITLKGITNG
GKDPCMDTFRTTTLPILKHFGVPMESLELKIVRRGAPPLGGGEVRLKVPMVPTSLTAVT
WMDEGMVKRVRGVAYSSRVSPQMSNRMVDSARGVLNPFLPDVYIFSDHVKGDEAGKS
PGYGISMVAETTTGCLLSAEGAALTQSHDEDEDTEKQEKAMLPEDIGSRAAMLLLEEIRE
GGVVDSTHQSLLFLLCALCPEDVSKIRVGKLSPHAILTLRHIKLILGVQFNIKPDPTTGTIILT
CVGSGYKNIFRKTS
>GpRCL1 (Gonium pectoral) KXZ50132.1
MLRFKGCQHFRQRLVCSTLSGRPIRIDDIRAADPESPGLRDFEASFLRLLEKLTNGCVVEI
NETGTSLRYRPGVVVCGMGLSHDCGTSRGIGYFLEPLLVLGLYGKKALSITLKGITNDST
DPGVDTFRTVTLPLMRKALGLEDGLELRVVSRGAAPGGGGEVVLRVPLVKELGPIKLVD
EGMVKRIRGVAFSMKVSPQITNRMVDGARGVLNSLLADVYVFTDATSGAASGKSPGYGI
TLVAETTSGCLISAEACHSAPRSQEDVAPQRDAGSAQQVPEDIGTTAAHLLLEEVRRGG
VVDGSHQGLMLLLCALGPEEANSLRLGPLTPHAVRTLRHIRDFFGVTFSLRTERTSNTLF
ATCVGANVRNVSRRVT
