# Supplementary material for: Maize RNA 3'-terminal phosphate cyclase-like protein promotes 18S pre-rRNA cleavage and is important for kernel development
Source: Plant Cell. 2022 Feb 15;34(5):1957–79. doi: 10.1093/plcell/koac052 (PMC9048941; doi:10.1093/plcell/koac052)
Supplement: koac052_supplementary_data [file koac052_supplementary_data.zip › tpc.21.00820_Supplemental Figures and Tables.pdf]

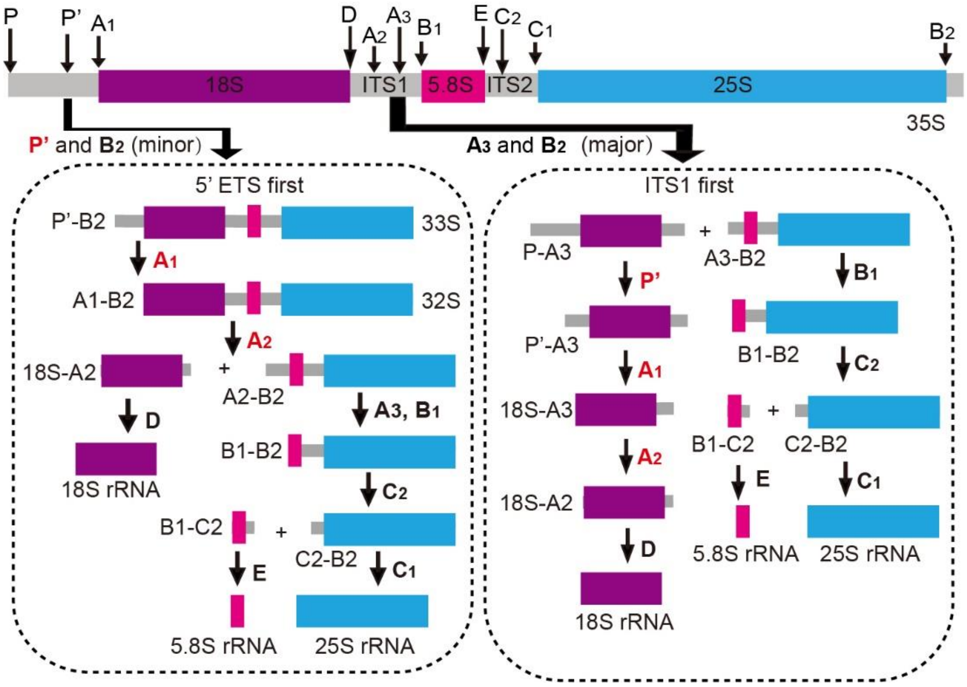

Supplemental Figure S1. Diagram showing general processing procedure and sites of pre-rRNA in plants. (Supports Introduction, Figure 8, Discussion)

Two alternative pathways process the 35S pre-rRNA transcribed from 45S rDNA: the minor 5'ETS-first pathway and the major ITS1- first pathway in Arabidopsis, rice and maize, characterized by the cleavage at P' or A_3 site in the first step, respectively (Hang et al., 2018; Saez-Vasquez and Delseny, 2019; Liu et al., 2020). In the major pathway, 35S pre-rRNA is subjected to cleavage at site A_3 in ITS1 and removal of 3' ETS at the B_2 site, producing P-A3 and A3-B2 intermediates. The P-A3 fragment is further processed at sites P' and A_1 of the 5′ ETS and the D and A_2 sites to form 18S rRNA. A3-B2 pre-rRNA fragment undergoes exoribonucleolytic trimming or endonucleolytic cleavages to form 5.8S and 25S rRNA. In the minor pathway, the 35S pre-rRNA is first processed at sites P' and B_2, further at A1, thus generating 33S and 32S intermediated in order. The endonucleolytic processing of 32S pre-rRNA at site A_2 within ITS1 generates 18S-A2 and A2-B2 intermediates. After cleavage at A_3, the A2-B2 fragment undergoes the same processing pattern as the major pathway to form 5.8S and 25S rRNA.

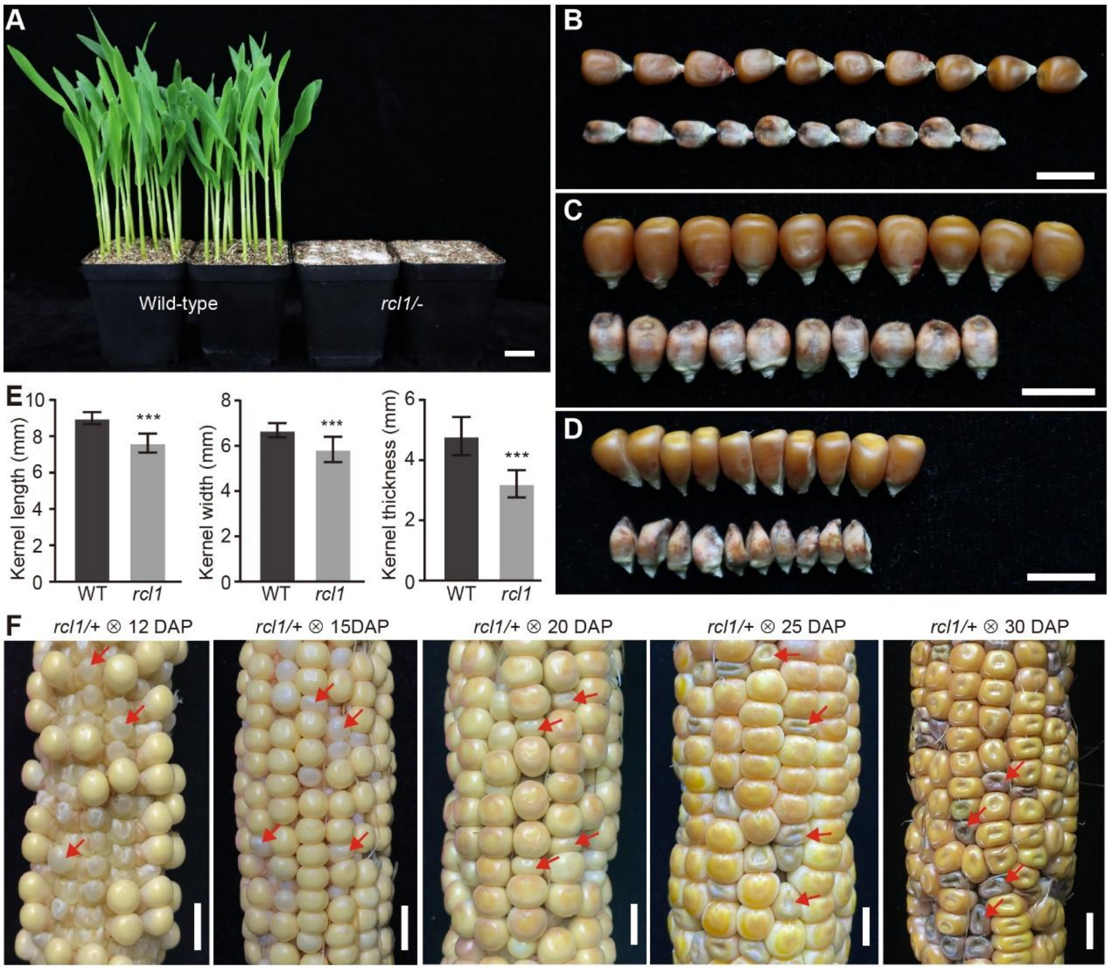

Supplemental Figure S2. Comparison of kernel phenotypes in WT and *rcl1* mutant. (Supports Figure 1)

(A) Germination of the WT and *rcl1* Seeds. Bar =1 cm.

(B) to **(D)** Comparison of kernel length **(B)**, kernel width **(C)** and kernel thickness **(D)** in the WT and *rcl1*. Bars = 1 cm.

(E) Quantitative measurement of kernel length (left), kernel width (middle) and kernel thickness (right) in the WT and *rcl1*. Error bars represent ± SD (n = 30). Three independent measurements using different ears were performed with similar results. ***P < 0.001, Student's *t* test.

(F) Developing ears harvested from heterozygous *rcl1/+* plant. The time was shown as indicated. Red arrows point to homozygous mutants. DAP, day after pollination. Bars = 1 cm.

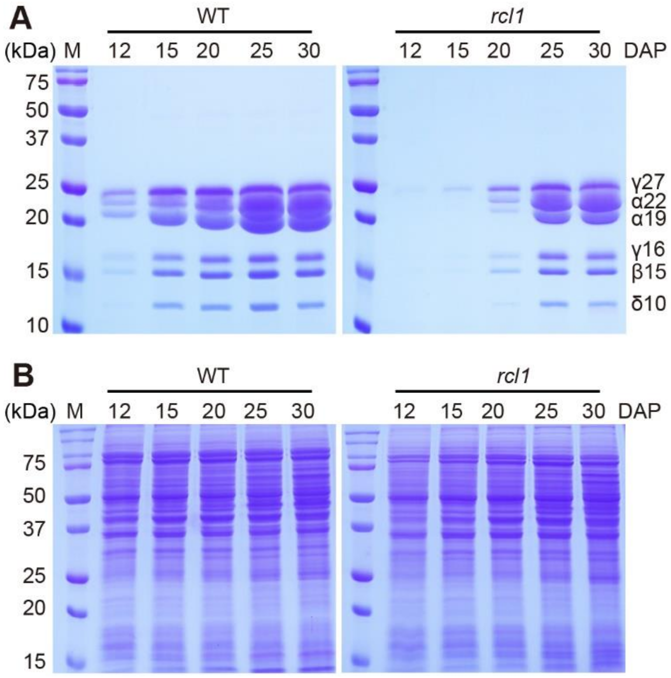

Supplemental Figure S3. Comparison of zeins and non-zein protein synthesis between the WT and *rcl1* developing Kernels. (Supports Figure 3)
(A) and (B) SDS-PAGE analysis of zein **(A)** and non-zein **(B)** from seeds of the WT and *rcl1* mutant. Fresh seeds at indicated stages were harvested for protein extraction. M, Standard protein marker. Molecular weight is labeled on the left side.

A

Gene ID	Chr	Start	End	Expression in B73 seeds						
				4 DAP	8 DAP	12 DAP	16 DAP	20 DAP	28 DAP	36 DAP
ZmMIR156j	7	134,663,764	134,663,888	0.00	0.81	1.27	0.20	0.00	0.00	0.49
Zm00001d020852	7	134,723,869	134,728,829	0.79	0.08	0.14	0.21	0.23	4.27	13.57
**Zm00001d020853**	**7**	**134,779,523**	**134,783,267**	**31.42**	**51.19**	**27.43**	**24.58**	**24.72**	**24.00**	**32.04**
Zm00001d020855	7	134,788,468	134,788,875	0.00	0.00	0.00	0.00	0.00	0.00	0.00
Zm00001d020856	7	134,790,920	134,791,488	0.00	0.00	0.00	0.00	0.00	0.00	0.00
Zm00001d020857	7	134,894,272	134,896,380	0.00	0.00	0.00	0.00	0.00	0.00	0.00
Zm00001d020858	7	134,901,536	134,904,779	5.39	4.67	3.32	4.75	5.01	10.14	12.89

B

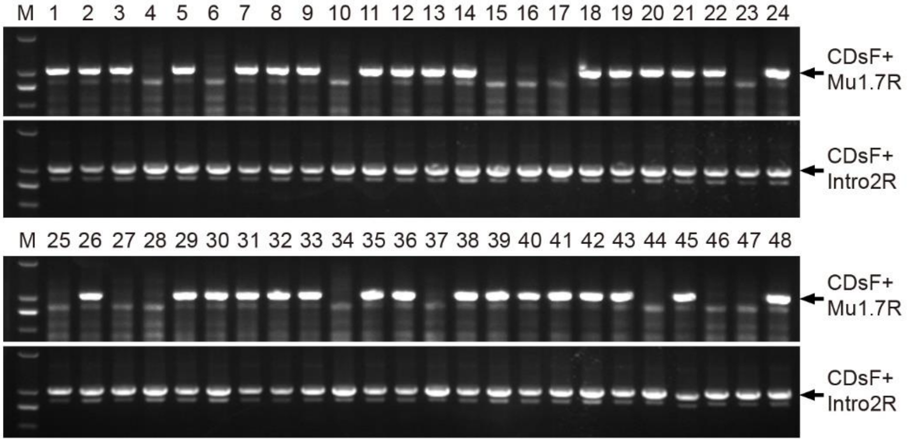

Supplemental Figure S4. Expression analysis of candidate genes and linkage analysis of Mu-1.7 insertion. (Supports Figure 4)

(A) The expression level of seven candidate genes in the B73 kernel. Candidate genes are located between indel marker Indel-134.66 and Indel-134.90. Expression data were obtained from RNA-Seq analysis (Chen et al., 2014).

(B) Genetic linkage analysis of Mu-1.7 and *rcl1* phenotype. The germinated seeds with normal wild-type phenotype were subjected to genotyping.

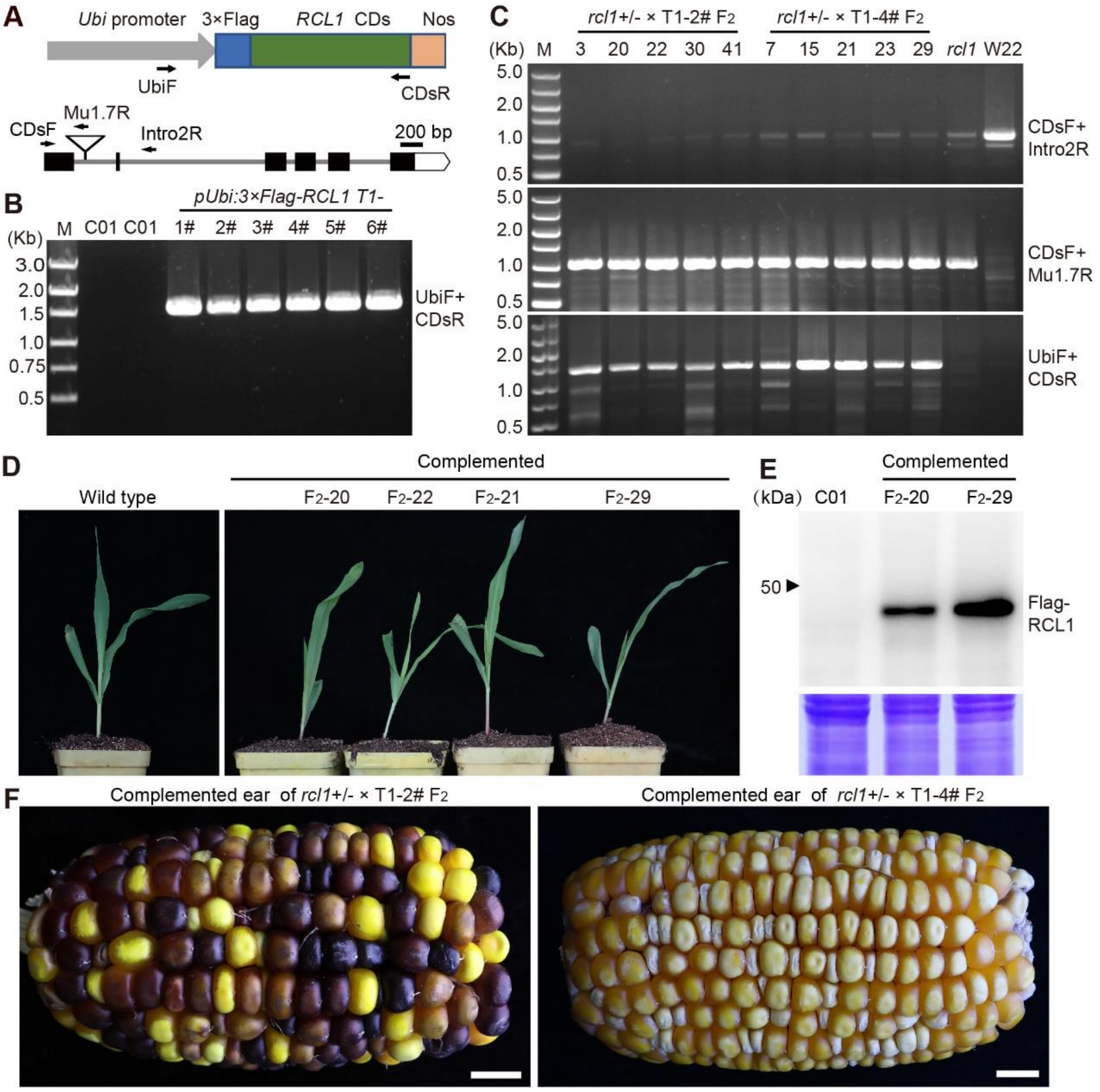

Supplemental Figure S5. Genetic complementation of *rcl1* mutant. (Supports Figure 4)
(A) Schematic diagram showing construction for over-expression of *RCL1* and primers used for genotyping.
(B) Identification of transgenic T1 plants using primers as indicated. The C01 inbred line was used as a negative control.
(C) Genotyping analysis of F_2 seedling harvest from the cross between *rcl1/+* and transgenic T1 plants. W22 inbred line (*rcl1* genetic background) and *rcl1* mutant were used as controls.
(D) Seedling of complemented homozygous *rcl1* showed similar growth compared to wild type.
(E) Immunoblotting detection of Flag-RCL1 protein in the kernels from self-pollinated complemented plants. The C01 inbred line was used as a negative control. The gel stained with Coomassie brilliant blue serves as the loading control.
(F) Images of complemented *rcl1* ears. Homozygous *rcl1* plants with Flag-RCL1 transgene produced normal kernels. Bars = 1 cm.

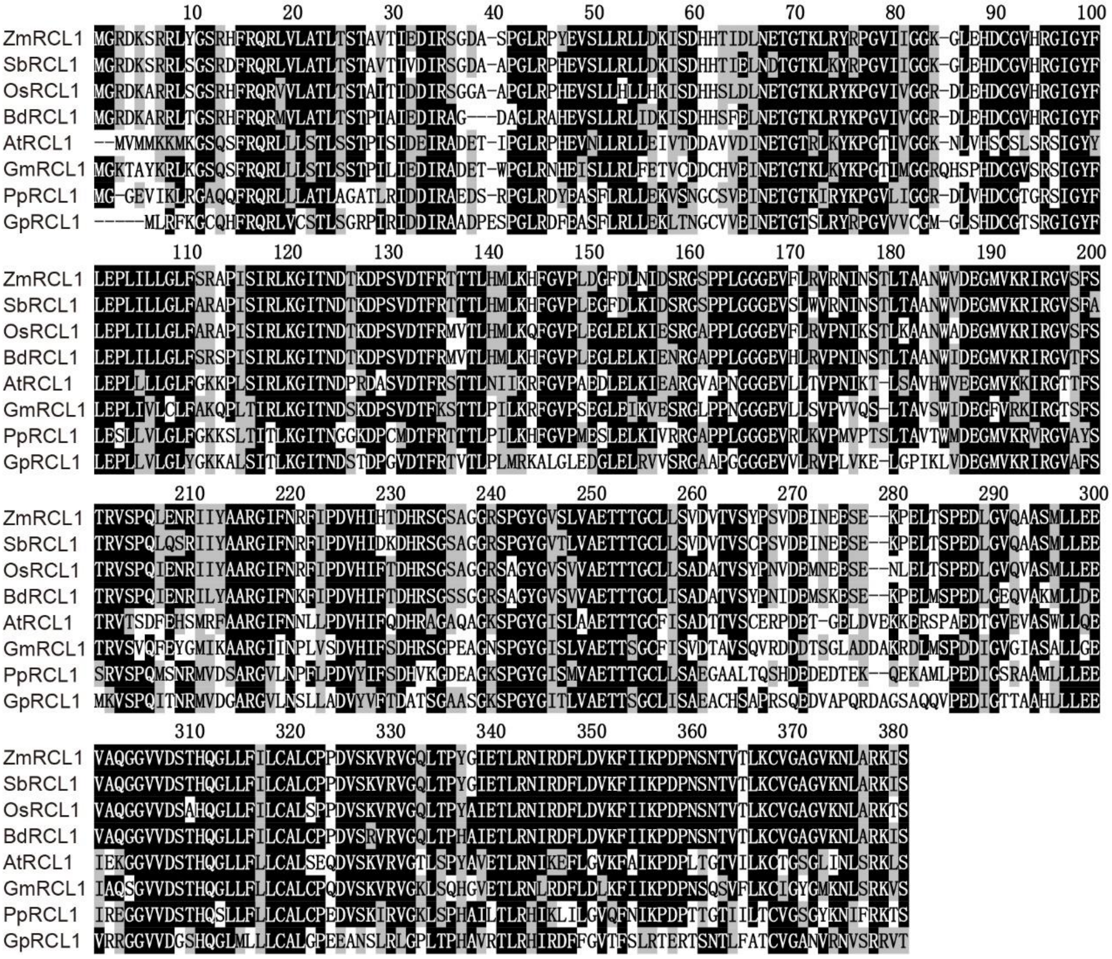

Supplemental Figure S6. Protein alignment of RCLs from representative plants. (Supports Figure 6)

Amino acid sequence of ZmRCL1 and its homologues from representative plant species. RCL proteins originate from the following organisms: ZmRCL1, *Zea mays* (NP_001130728.1); SbRCL1, *Sorghum bicolor* (XP_002460403.1); OsRCL1, Oryza sativa (XP_015628085.1); BdRCL1, *Brachypodium distachyon* (XP_003563054.1); AtRCL1, *Arabidopsis thaliana* (NP_680196.1); GmRCL1, *Glycine max* (NP_001242399.1); PpRCL1, *Physcomitrium patens* (XP_024397674.1); GpRCL1, *Gonium pectoral* (KXZ50132.1). The alignment was performed using the ClustalW program (Thompson, et al., 1994). The black and grey boxes indicate identical and conserved amino acids, respectively, in a threshold of 70%.

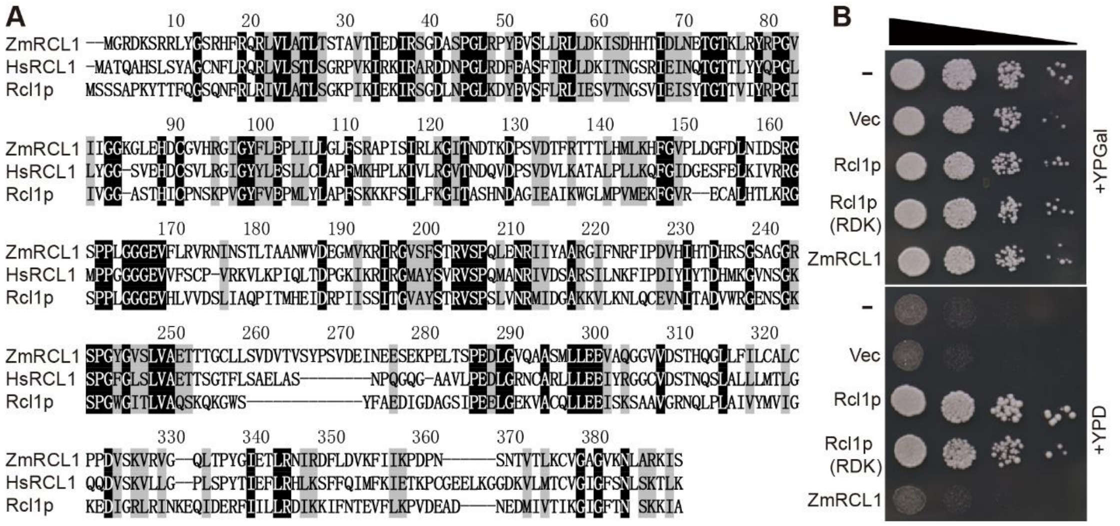

Supplemental Figure S7. ZmRCL1 is a homolog of Rcl1p, but cannot complement its function. (Supports Figure 6)

(A) Amino acid alignment of 3' RNA phosphate cyclase-like (RCL) proteins from *Zea mays* (ZmRCL1), human (HsRCL1) and yeast (Rcl1p). The alignment was performed using the ClustalW program (Thompson et al., 1994). The black and grey boxes indicate identical and conserved amino acids, respectively, in a threshold of 100%.

(B) Gradient dilutions of *S. cerevisiae* strain YKK55 on YPGal and YPD media with different transformations as indicated. Genomic Rcl1p in YKK55 was replaced by the galactose-inducible promoter (Horn et al., 2011). Transformations were performed with plasmids vector (Vec), yeast Rcl1p, point mutations at R327A, D328A, and K330A (RDK) and ZmRCL1 as indicated on the left. The complementation ability of ZmRCL1 was determined by comparison of yeast growth on galactose (YPGal) and glucose (YPD) solid media, respectively. Short dashes (-) indicate no transformation.

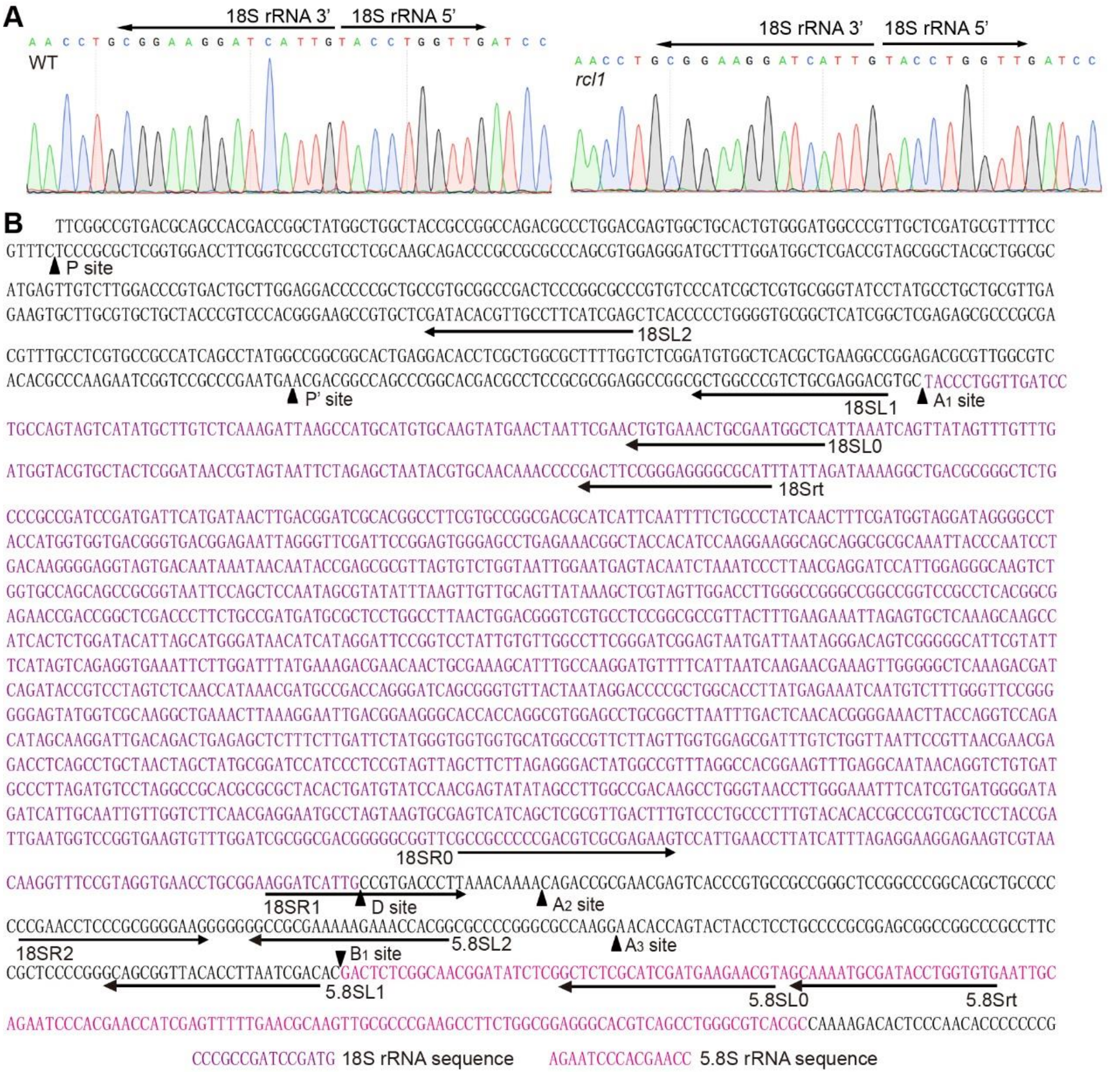

Supplemental Figure S8. Sequencing and processing sites around the 18S rRNA. (Supports Figure 7, Figure 8)

(A) Sequencing of the circular RT-PCR products. The intact sequence of mature 18S rRNA can be detected in *rcl1* and WT.

(B) The sequence and processing sites around 18S rRNA (Liu et al., 2020). Wedge shapes pointed to the precise cleavage sites. Long arrowheads showed the primers used for circular RNA reverse transcription and RT-PCR amplification.

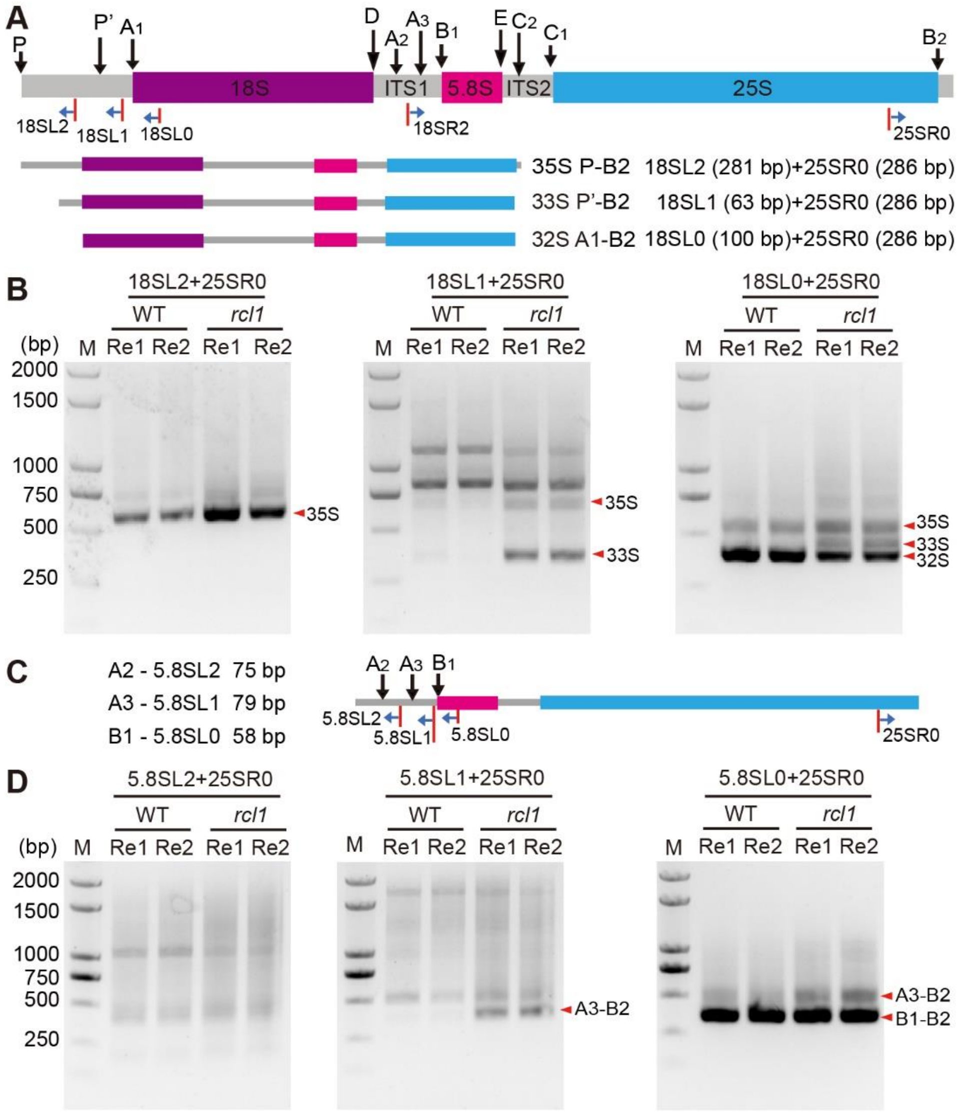

Supplemental Figure S9. Primers and gel pictures of circular RT-PCR. (Supports Figure 8)
(A) Diagram illustrating various primer pairs used for amplification of 5' EST first pathway intermediates by circular RT-PCR.
(B) Agarose gel of circular RT-PCR using primers as indicated in **(A)**. Target fragments are labeled on the right.
(C) Diagram illustrating various primer pairs used for amplification of 5.S and 25S pre-rRNAs by circular RT-PCR.
(D) Agarose gel of circular RT-PCR using primers as indicated in **(C)**. Target fragments are labeled on the right.
In B and D, Re1 and Re2 represent two biological replicated samples from different ears. M, DNA marker.

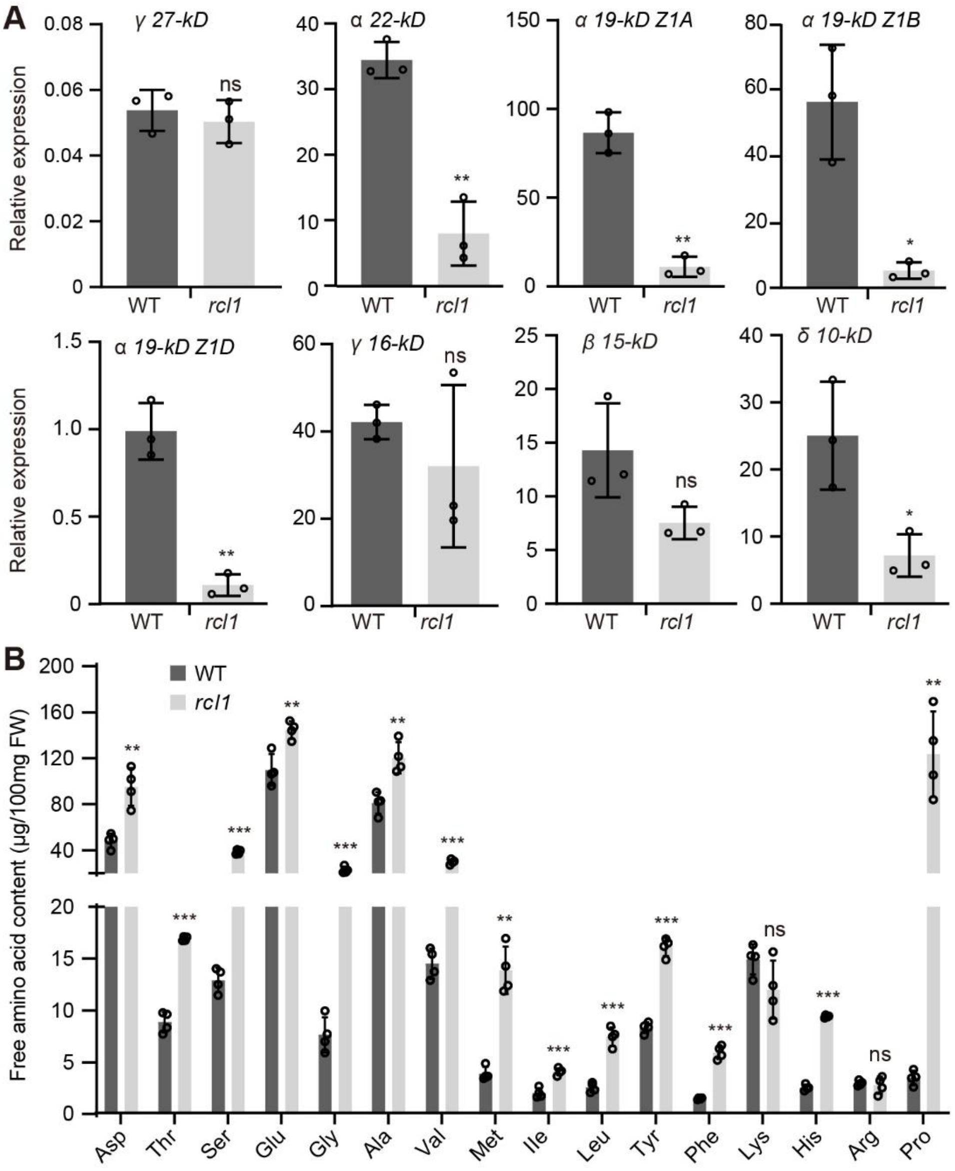

Supplemental Figure S10. Expression of zein encoding genes and measurement of free amino acid in WT and *rcl1* endosperm at 15 DAP. (Supports Figure 9)

(A) Quantitative RT-PCR analysis of the zein encoding genes in 15-DAP endosperm from WT and *rcl1*. The maize *Ubi* gene was used as an internal control.

(B) Measurement of free amino acid in the WT and *rcl1* mature kernels.

Error bars represent ± SD calculated from three (A) or four (B) biological replicated samples from different ears. *P < 0.05, **P < 0.01, ***P < 0.001, ns, no significant. Student's *t* test.

Supplemental Table S1. Relative rRNA levels (% of total area) in wild type (WT), *rcl1* and *rcl1-C*. (supports Figure 7)

	WT		*rcl1*		*rcl1-C*		25S/18S			Fold-change *rcl1*/WT
	25S	18S	25S	18S	25S	18S	WT	*rcl1*	*rcl1-C*	
Re1	42.1	21.2	51.2	12.3	29.1	15.7	1.986	4.163	1.853	2.096
Re2	41.3	21.5	52.5	13.1	30.7	16.3	1.921	4.008	1.883	2.086
Re3	40.3	20.5	51.7	12.2	31.7	16.2	1.966	4.238	1.956	2.156

Total RNA of three replicates (Re1 to Re3) from different ears were analyzed.

Supplemental Table S2. Measurement of free amino acids content in the endosperm from wild type and *rcl1*. (supports Figure 9)

Amino acid name	Free amino acids Content of 15 DAP endosperm (μg/100mg)								Average (WT)	Average (*rcl1*)	Fold of Change (*rcl1*/WT)	*P*-value
	WT				*rcl1*							
	Re1	Re2	Re3	Re4	Re1	Re2	Re3	Re4				
Asp	49.03	54.51	39.73	50.13	112.9	74.74	91.14	102.01	48.35±5.38	95.20±14.10	1.97	<0.01**
Thr	9.63	9.77	8.35	7.74	17.14	16.75	16.76	17.1	8.87±0.86	16.94±0.18	1.91	<0.001***
Ser	13.7	14.04	12.52	11.48	36.69	39.95	37.07	40.65	12.93±1.01	38.59±1.73	2.98	<0.001***
Glu	107.59	128.94	106.56	95.99	144.01	152.52	134.55	147.17	109.77±11.96	144.56±6.53	1.32	<0.01**
Gly	7.02	9.97	7.72	5.91	20.74	22.79	21.18	27.11	7.66±1.49	22.95±2.52	3	<0.001***
Ala	83.08	90.39	82.59	68.04	108.58	139.29	121.57	112.53	81.03±8.11	120.49±11.83	1.49	<0.01**
Val	15.45	16.02	13.73	12.91	29.44	27.18	30.46	32.54	14.52±1.26	29.90±1.93	2.06	<0.001***
Met	4.89	3.66	3.66	3.43	11.87	14.28	16.95	12.42	3.91±0.57	13.88±1.99	3.55	<0.01**
Ile	2.69	2.02	1.75	1.64	4.06	3.66	4.49	4.15	2.03±0.41	4.09±0.29	2.02	<0.001***
Leu	3.03	2.85	2.31	2.06	7.49	6.3	7.7	8.47	2.56±0.39	7.49±0.78	2.92	<0.001***
Tyr	8.9	8.5	8.39	7.63	14.91	16.53	16.2	16.93	8.35±0.46	16.14±0.76	1.93	<0.001***
Phe	1.58	1.46	1.38	1.56	5.69	5.21	6.34	6.64	1.49±0.08	5.97±0.56	3.99	<0.001***
Lys	15.23	16.35	15.28	12.92	9.02	15.66	12.43	10.95	14.95±1.25	12.02±2.43	0.8	0.1288
His	2.95	2.55	2.47	2.24	9.38	9.55	9.27	9.46	2.55±0.26	9.41±0.10	3.69	<0.001***
Arg	3.29	3.05	2.88	2.73	1.74	3.23	2.53	3.62	2.98±0.21	2.78±0.71	0.93	0.6585
Pro	2.6	3.48	3.6	4.31	84.02	169.37	105.37	135.16	3.50±0.61	123.48±32.13	35.31	<0.01**

Four biological replicated samples (Re1 to Re4) from different ears were analyzed. **P < 0.01 and ***P < 0.001, Student's *t* test.

Supplemental Table S3. Primers used in this study.

Primer name	Sequences (5'-3')	Usage
Indel-83.1F	GAGGATGGCTGTCGGATTT	Mapping-based cloning
Indel-83.1R	CCCTCTGATTTTGAGGGACA	
Indel-112.3F	ATGGAGGCTTATGGCTACGA	
Indel-112.3R	TCGAGAGGTCGTAACCGTTTGC	
Indel-125.25F	CTGCAAACAAGGCTGTCTCA	
Indel-125.25R	AGGACACGCATCAGAAGCCATG	
Indel-129.77F	TATTCCAGCAGTGACAACGC	
Indel-129.77R	CGAATATAAGCGGTGCCCTA	
Indel-134.26F	CAACAAGAAATAGGTGGAACCT	
Indel-134.26R	CAGATGCGAGCAGCAGTAAG	
Indel-134.66F	AAGAATGGCACAGTATTTCCCC	
Indel-134.66R	AAGCCTCGCTTCTGCGAGGGCC	
Indel-134.90F	GTCATGATCCGATCCTTTGTAC	
Indel-134.90R	CCATGGCAGACAGGCGGTTGTG	
Indel-136.2F	GCTGCTACGAATAACCCGAA	
Indel-136.2R	TGTGCCGTGTGGTGTACTACG	
Indel-147.9F	GGTTGCAGCTACGTACCCAACC	
Indel-147.9R	TGGCCGAACGAGCACGACATGG	
Indel-152.6F	GCAGTCTTGTTGCGTCTGAA	
Indel-152.6R	CATGCCCGATTTTGAGAGAT	
Indel-167.9F	AGCTGCATGGCAGGAGTATT	
Indel-167.9R	GAGTCCTCGACATTCTTCGC	
RCL1subF	gagctcATGGGGCGAGACAAGAGCCGTC	Subcellular localization
RCL1subR<br>CDsR	gtcgacTGAAATCTTCCGAGCAAGATTC	Subcellular localization/<br>Transgene identification
RRP7subF	GGTACCATGAAGGAGACGAGAAAGCTGA	Subcellular co-localization
RRP7subR	TCTAGAGAAAGGCTTAAACCTACGAGCA	
18Srt	ATGCGCCCCTCCCGGAAGTC	Circular RT-PCR
5.8Srt	CACCAGGTATCGCATTTTGC	
18SL0	GAGCCATTCGCAGTTTCACAG	
18SL1	CGTCCTCGCAGACGGGCCAGC	
18SL2	CTCGATGAAGGCAACGTGTATC	
18SR0	AGGAAGGAGAAGTCGTAACAAG	
18SR1	AGGATCATTGCCGTGACCCTT	
18SR2	CCGAACCTCCCGCGGGGAAG	
25SR0	GGATTATGACTGAACGCCTCTAAG	
5.8SL0	ATTTTGCTACGTTCTTCATCG	
5.8SL1	GTCGATTAAGGTGTAACCGCTGC	
Ubi1qRTF	CTGGTGCCCTCTCCATATGG	RT-qPCR
UBI1qRTR	CAACACTGACACCGACTCATGACA	
RCL1CDsF<br>RCL1qRTF	ATGGGGCGAGACAAGAGCCGTC	Genotype identification/RT-qPCR
RCL1qRTR	CCCTCGAAAATAGACCAAGCAG	RT-qPCR
Intro2R	GAATGCACTCGATTTCGGTCAG	Genotype identification
Mu1.7R	CGACGTCTTCGAGTGCTGGCTC	
UbiF		
RCL1-pamF	ACCATTGACCTCAACGAGACgttttagagctagaaatagc	Genome editing
RCL1-pamR	GTCTCGTTGAGGTCAATGGTAATTCGGTGCTTGCGGCTCG	
RCL1 - FlagF	CGACTCTAGAGGATCCATGgactacaaagaccatgacggtgattataa<br>agatcatgatatcgattacaaggatgacgatgacaagGGCGGCGGCGGC<br>GCCGCCATGGGGCGAGACAAGAGCCG	Genetic complementation
RCL1 - FlagR	GGGGAAATTCGAGCTCTTATGAAATCTTCCGAGCAAG	
RCL1antiF	ATGGGGCGAGACAAGAGCCG	In situ hybridization
RCL1antiR	TAATACGACTCACTATAGGGCGAATTATGAAATCTTCCGA<br>GCAA	
RCL1senseF	TAATACGACTCACTATAGGGCGAAATGGGGCGAGACAAG<br>AGCCG	
RCL1senseR	TTATGAAATCTTCCGAGCAA	
AL9 anti F	CGTCCCCCAAGAACTAGTAAAC	
AL9 anti R	TAATACGACTCACTATAGGGCGAACCCTCTTGCGATATCA<br>TCAGTT	
AL9 sense F	TAATACGACTCACTATAGGGCGAACGTCCCCCAAGAACTA<br>GTAAAC	
AL9 sense R	CCCTCTTGCGATATCATCAGTT	
Mn1 anti F	ATGTACTACAAGGGGTGGTACC	
Mn1 anti R	TAATACGACTCACTATAGGGCGAATCAGGCCCCGTTCATG<br>ACCGGC	
Mn1 sense F	TAATACGACTCACTATAGGGCGAAATGTACTACAAGGGGT<br>GGTACC	
Mn1 sense R	TCAGGCCCCGTTCATGACCGGC	

Supplemental Table S3. Continued

P1	AGCGTAGCCGCTACGGTCGAGCCA	
P2	ACGTCCTCGCAGACGGGCCAGC	
P18S	CATATGACTACTGGCAGGATCAACC	
P4	TCTGTTTTGTTTAAGGGTCACGG	RNA gel blot probes
P5	GGCAGCGTGCCGGGCCGGAGC	
P5.8S	GCAATTCACACCAGGTATCGCATTT	
P25S	TCCTCGTAAGTTTCTTCTCCTCCGC	
27kD-F	TGCCTACAGCCGTCTCG	
27kD-R	GAGGGCAACGAGCAACAC	
22kD-F	TTCCACAATGCTCACTTGCT	
22kD-R	GTTGTTGTAAGACGCTCGCC	
19kD-AF	GCTCCTTGGTCTTTCTGCAA	
19kD-AR	GGTAACTGCTGTAATAGGGCTGATG	
19kD-BF	CCAGCCCTATCTTTGGTGCA	
19kD-BR	TCAGTGCGGCCAATTGGTTA	
19kD-DF	GCACAACAACTACAACAACA	
19kD-DR	AATGGTAGTAGCTGTTGTGC	
16kD-F	CGGCGGTGTCTACTACTGAG	
16kD-R	GGTTCATTCAGGTCATTGCTC	
15kD-F	CTACCGCACCAACCCCTG	
15kD-R	TCAAGCGGCCGATAGATTC	
10kD-F	AGATGATGACGCCTAACA	
10kD-R	ATGAATGGTAACTGCTG	
SS3aqF	TGTCAACCTGGCGAATAAGC	
SS3aqR	GGCTCGTTCCTTGTCATTGTC	
SS1qF	GTCTGCTTTGGCTGCCTTG	
SS1qR	AGGACAACAACACAGGTAATAATC	
SS2aqF	ATCGTGGTGGCTGCTGAATG	
SS2aqR	GTTCACTTCTAGGTCCTGTCCTGC	
GBSS1qF	GTCGAAGGCGAGGAGATC	
GBSS1qR	CGCTTATTAGGTTGTGCCA	
Sh1qF	TGTTTCACCGCAATTCGCA	
Sh1qR	AGACAGGTGAACGAGCAGGC	
Bt2qF	ACTAATGGGTGCGGACTACTATG	
Bt2qR	TACCCGTCTGTCTCCATTGC	RT-qPCR
Sh2qF	TGGGAGCGGACACCTATG	
Sh2qR	TCACCACGATTCCAGACCTT	
SBE2bqF	CGAAAGCCTGGGGTGTAT	
SBE2bqR	CACTGGAGCATAGACGACACAT	
SBE1qF	TGAAGGGGTGCCAGGG	
SBE1qR	GCCTCCTTGTCTTCTTTGCTAC	
Su1qF	CGGTGGTTGTTGGGCTTC	
Su1qR	CGCAATACAAGGATGATGGAG	
PGD1qF	CTACCGCAGGGACAGGCT	
PGD1qR	TTACAAAGATCAATAGACAAGTTCC	
PGD2qF	ACACGTACGAGAGGGTTGAC	
PGD2qR	AATGGGAGAGACTTTGGACTT	
PGD3qF	TAGCACGCCAGGAATGACT	
PGD3qR	TAACCCACAGAGAGAAATCACAC	
PPDK1 qF	GAAGGCTGGGCTGGATTAC	
PPDK1 qR	AAAGGGAGATGGGATTGTAGC	
PPDK2 qF	CAGGGATGATGTGGGGAAG	
PPDK2 qR	CGTAATCCAGCCCAGTCTTG	
O2qF	CTGGGGTTCAAGATGCCTAC	
O2qR	TGGTTCAGAGCGGCAATG	
OHP1qF	CAGGCGGGGGATCAAGTC	
OHP1qR	TGTGCCTCCAGTTCATTCAAG	
OHP2qF	CGGATGCAACAACGGCTAC	
OHP2qR	CGTTAGGCACCAGGGACT	
PBFqF	GTTAGTGTGCCAGACCGTG	
PBFqR	GCTTACTGCAAATGAACTCTC	
UbiqF	CTGGTGCCCTCTCCATATGG	
UbiqR	CAACACTGACACCGACTCATGACA	

Supplemental references

Chen, J., Zeng, B., Zhang, M., Xie, S., Wang, G., Hauck, A., and Lai, J. (2014). Dynamic transcriptome landscape of maize embryo and endosperm development. Plant Physiol **166,** 252-264.

Hang, R., Wang, Z., Deng, X., Liu, C., Yan, B., Yang, C., Song, X., Mo, B., and Cao, X. (2018). Ribosomal RNA Biogenesis and Its Response to Chilling Stress in Oryza sativa. Plant Physiol **177,** 381-397.

Horn, D.M., Mason, S.L., and Karbstein, K. (2011). Rcl1 protein, a novel nuclease for 18 S ribosomal RNA production. J Biol Chem **286,** 34082-34087.

Liu, G.Q., Yan, P.S., Du, Q.G., Wang, Y.F., Guo, Y., Fu, Z.Y., Wang, H.Q., and Tang, J.H. (2020). Pre-rRNA processing and its response to temperature stress in maize. Journal of Experimental Botany **71,** 1363-1374.

Saez-Vasquez, J., and Delseny, M. (2019). Ribosome Biogenesis in Plants: From Functional 45S Ribosomal DNA Organization to Ribosome Assembly Factors. Plant Cell **31,** 1945-1967.

Thompson, J.D., Higgins, D.G., and Gibson, T.J. (1994). CLUSTAL W: improving the sensitivity of progressive multiple sequence alignment through sequence weighting, position-specific gap penalties and weight matrix choice. Nucleic Acids Res **22,** 4673-4680.
